# Alteration of the late endocytic pathway in Charcot–Marie–Tooth type 2B disease

**DOI:** 10.1007/s00018-020-03510-1

**Published:** 2020-04-13

**Authors:** Roberta Romano, Cristina Rivellini, Maria De Luca, Rossana Tonlorenzi, Raffaella Beli, Fiore Manganelli, Maria Nolano, Lucio Santoro, Eeva-Liisa Eskelinen, Stefano C. Previtali, Cecilia Bucci

**Affiliations:** 1 Department of Biological and Environmental Sciences and Technologies (DiSTeBA), University of Salento, Lecce, Italy; 2 Institute of Experimental Neurology (INSPE), IRCCS San Raffaele Scientific Institute, Milan, Italy; 3 Department of Neurosciences, Reproductive Sciences and Odontostomatology, University of Naples “Federico II”, Naples, Italy; 4 Salvatore Maugeri Foundation, Institute of Telese Terme, Benevento, Italy; 5 Institute of Biomedicine, University of Turku, Turku, Finland; 6 Molecular and Integrative Biosciences Research Programme, University of Helsinki, Helsinki, Finland

**Keywords:** RAB7A, Endocytosis, Lysosome, RAC1, Migration, EGFR

## Abstract

The small GTPase RAB7A regulates late stages of the endocytic pathway and plays specific roles in neurons, controlling neurotrophins trafficking and signaling, neurite outgrowth and neuronal migration. Mutations in the *RAB7A* gene cause the autosomal dominant Charcot–Marie–Tooth type 2B (CMT2B) disease, an axonal peripheral neuropathy. As several neurodegenerative diseases are caused by alterations of endocytosis, we investigated whether CMT2B-causing mutations correlate with changes in this process. To this purpose, we studied the endocytic pathway in skin fibroblasts from healthy and CMT2B individuals. We found higher expression of late endocytic proteins in CMT2B cells compared to control cells, as well as higher activity of cathepsins and higher receptor degradation activity. Consistently, we observed an increased number of lysosomes, accompanied by higher lysosomal degradative activity in CMT2B cells. Furthermore, we found increased migration and increased RAC1 and MMP-2 activation in CMT2B compared to control cells. To validate these data, we obtained sensory neurons from patient and control iPS cells, to confirm increased lysosomal protein expression and lysosomal activity in CMT2B-derived neurons. Altogether, these results demonstrate that in CMT2B patient-derived cells, the endocytic degradative pathway is altered, suggesting that higher lysosomal activity contributes to neurodegeneration occurring in CMT2B.

**Electronic supplementary material:**

The online version of this article (10.1007/s00018-020-03510-1) contains supplementary material, which is available to authorized users.

## Introduction

Endocytosis is an extremely regulated process by which cells internalize different plasma membrane components and molecules from the extracellular space [1]. This pathway plays a role in several cellular events, including termination of signaling pathways by targeting signaling receptors to degradation compartments such as late endosomes and lysosomes [1, 2]. Transport to late endosomes and lysosomes is finely controlled by sequential events and by several cellular proteins, including RAB GTPases, molecular motors and sorting regulators that altogether assure correct shipment to compartments [1, 3].

RAB7A, hereafter called RAB7, a ubiquitous RAB GTPase, regulates clustering and fusion of late endosomes and lysosomes in the perinuclear area, controlling biogenesis of and transport to lysosomes, phagolysosomes and autolysosomes [4–8]. Interestingly, several studies demonstrate that RAB7 plays specific roles in neurons, controlling axonal retrograde transport of neurotrophins, neurotrophin receptor signaling, neurite outgrowth and the final phase of immature cortical neuron migration [9–14]. Furthermore, RAB7 interacts with and controls assembly of two intermediate filament proteins, peripherin and vimentin, which play an essential role in neurite outgrowth and axonal regeneration, and which also regulate the degradative endocytic pathway, providing positional information for late endocytic organelles and being responsible for their function [15–22].

Notably, a number of studies show that alterations of endocytic traffic and axonal transport are causative of several neurodegenerative diseases, such as Alzheimer’s disease, Huntington’s disease and amyotrophic lateral sclerosis [23]. In line with these findings, five missense mutations in the RAB7 gene cause Charcot–Marie–Tooth type 2B (CMT2B) disease, a rare inherited autosomal dominant neuropathy affecting the peripheral nervous system [24–27].

CMT2B is one of the numerous forms of CMT disease. It is an axonal neuropathy, characterized by progressive distal weakness and atrophy, accompanied by sensory loss and ulcero-mutilating complications [28–32]. CMT2B is a rare form of CMT and only one Italian family carrying the RAB7^V162M^ mutation has been identified up to now [33].

The biochemical properties of four CMT2B-causing mutants (RAB7^L129F^, RAB7^K157N^, RAB7^N161T^ and RAB7^V162M^) have been previously investigated, demonstrating that these mutants show higher nucleotide *K*_off_ compared to the wild-type protein, and, therefore, release nucleotides faster [34–36]. As in the cell GTP concentration is higher than GDP, CMT2B mutants are mostly in the GTP-bound form, and were initially predicted to be active mutants [36]. However, these mutant proteins also release GTP faster than the wt protein, thus displaying a reduced GTPase activity per binding event [34–36], and, for this reason, they could inhibit activation of specific effectors while inducing activation of others. This hypothesis is corroborated by data in Drosophila, where neurodegeneration occurring in CMT2B is due to partial loss of function, and in zebrafish where axon growth and guidance defects are caused by a gain of function mechanism [37–39]. Therefore, CMT2B-causing mutations are neither "loss of function" nor "gain of function", but they behave as either inhibitory or activating, depending on kinetic requirements of the processes controlled by RAB7.

Importantly, the expression of CMT2B-causing RAB7 mutants alters several neuronal processes, modifying neurotrophin trafficking and, as a consequence, neurotrophin signaling pathways, thus resulting in inhibition of neurite outgrowth [40–42].

In this work, we analyzed different aspects of the endocytic pathway in skin fibroblasts and iPS-derived sensory neurons. Interestingly, we found that patient-derived CMT2B cells display greater expression of lysosomal markers as well as higher lysosomal activity compared to control cells, suggesting that these alterations could contribute to neurodegeneration.

## Materials and methods

### Cells and reagents

Dermal fibroblasts derived from two healthy individuals (control fibroblasts) and from three CMT2B patients belonging to the same Italian family carrying the RAB7^V162M^ mutation were obtained as previously described [43]. After informed consent for pathological diagnosis, samples were anonymously encoded to protect patient confidentiality and used under protocols approved by the Azienda Ospedaliera Universitaria "Federico II" Ethics Committee (*Ethical Committee Approval Protocol # 107/05*). Fibroblasts were cultured in Dulbecco's modified Eagle's medium (DMEM) supplemented with 15% fetal bovine serum (FBS), 2 mM l-glutamine, 100 U/ml penicillin and 10 mg/ml streptomycin in a 5% CO_2_ incubator at 37 °C. Chemicals and tissue culture reagents were from Sigma-Aldrich (St-Louis, MO, USA). HeLa cells were maintained in DMEM supplemented with 10% FBS, 2 mM l-glutamine, 100 U/ml penicillin and 10 mg/ml streptomycin in a 5% CO_2_ incubator at 37 °C.

### Antibodies

Primary antibodies used in this study were: mouse monoclonal anti-RAB7 (1:500, sc-376362), anti-RAB9 (1:200, sc-74482), anti-β-actin (1:1000, sc-47778), anti-RACGAP1 (1:200, sc-271110), anti-OCT3/4 (1:200; sc-5279) and anti EAP30 (1:200, sc-100892), rabbit polyclonal anti-RAB4 (1:200, sc-312), anti-RAB5 (1:200, sc-309), anti-ERK1 (1:200, sc-93), anti-HA (1:100, sc-805) and anti-pAKT (1:200, sc-7985-R), goat polyclonal anti-cathepsin D (1:500, sc-6486) from Santa Cruz Biotechnology, (Dallas, TX, USA); mouse monoclonal anti-tubulin (1:10,000, T5168) and anti-Smooth Muscle Actin (1:200; A2547) from Sigma-Aldrich (St. Louis, MO, USA); mouse monoclonal anti-cytokeratin CK8/18 (1:400; NCL-L-5D3) from Leica (Wetzlar, Germany); mouse monoclonal anti-LAMP2 (1:5000, H4B4), developed by J.T. August and J.E.K. Hildreth and obtained from the Developmental Studies Hybridoma Bank, (Iowa City, IA 52242); rabbit polyclonal anti-CI-MPR (1:2000, ab32815), anti-TSG101 (1:500, ab30871) anti-LAMP1 (1:4000, for immunoblot analysis and 1:1000 for immunofluorescence analysis, ab24170) and goat polyclonal anti-CGRP (1:200; ab36001) from Abcam (Cambridge, UK); rabbit polyclonal anti-peripherin (1:100, AB1530) and anti-Brn3a (1:100; AB5945) from Merck Millipore (Burlington, MS, USA); sheep polyclonal anti-TGN46 (1:500, AHP500) from Bio-Rad (Hercules, CA, USA); sheep polyclonal anti EGFR (1:1000, 20-ES04) from Fitzgerald (North Acton, MS, USA); rabbit monoclonal anti-AKT (1:1000, 4691) and phospho-p44/42 (Erk 1/2) (1:2000, 4370) from Cell Signaling Technology (Leiden, The Netherlands); mouse monoclonal anti-RAC1 (1:600, ARC03) from Cytoskeleton (Denver, CO, USA); rabbit polyclonal anti-Nanog (1:100; RCAB0002P-F) from ReproCELL (Glasgow, UK); rabbit polyclonal anti-Pax-6 (1:100; PRB-278P) from Covance (Princeton, NJ, USA); chicken polyclonal anti-Neurofilament M (1:1000; 822701) from BioLegend (San Diego, CA, USA); rabbit polyclonal anti-ARHGEF6 (1:1000; A302-558A) from Bethyl Laboratories (Montgomery, TX, USA). Secondary antibodies conjugated to fluorochromes (used at 1:600 dilution) or HRP (used at 1:5000 dilution) were from Invitrogen (Carlsbad, CA, USA), Fitzgerald, SouthernBiotech (Birmingham, AL, USA) or Jackson ImmunoResearch (Cambridgeshire, UK).

### Plasmids

Plasmids encoding HA-tagged RAB7^WT^ and the CMT2B-causing RAB7^V162M^ mutant protein have been previously described [36]. The pcDNA3-HA plasmid was constructed by inserting a DNA sequence coding for a 2xHA-tag into the KpnI restriction site of the pCDNA3.1 (Invitrogen, V79020) and it was used as empty vector in control transfections.

### Induced pluripotent stem cells generation

iPSCs were generated reprogramming fibroblasts from healthy controls and CMT2B patients carrying the RAB7^V162M^ mutation by Sendai viral transduction of the transcription factors OCT4, SOX2, KLF4, and c-MYC (CytoTune2.0-iPS Sendai Reprogramming Kit, Thermo Fisher, Waltham, MS, USA). iPSCs were then tested for Sendai virus clearance (CytoTune2.0-iPS Sendai Reprogramming Kit, Thermo Fisher) and pluripotency (Trilineage differentiation Kit, Stem Cell Technologies, Vancouver, Canada). Cells were regularly screened and confirmed negative for mycoplasma during both maintenance and differentiation (MycoAlert kit, Lonza, Basel, Switzerland).

### Induced pluripotent stem cells maintenance and differentiation in dorsal root ganglia (DRG) sensory neurons

iPSCs were maintained in feeder-free conditions using mTeSR1 medium (Stem Cell Technologies) on hES qualified Matrigel (Corning, Corning, NY, USA) coated plates. At 80–90% of confluency, iPSCs were passaged using 0.5 mM EDTA in PBS (Sigma-Aldrich) or ReLeSR (Stem Cell Technologies), in the presence of ROCK inhibitor (Stem Cell Technologies). Prior to differentiation, iPSCs were single cell dissociated with Accumax (Sigma-Aldrich) and plated onto hES qualified Matrigel coated six-well plates in the presence of ROCK inhibitor. Cells were grown in mTeSR1 medium for 24 h, then shifted to MEF-CM (Mouse embryo fibroblasts-conditioned medium) produced according to Lee et al. [44], supplemented with 10 ng/ml bFGF (Thermo Fisher). At approximately 40–50% of confluency (usually 24/48 h after the shift to CM medium), differentiation was started [45, 46]. The medium was replaced with KSR medium high glucose DMEM (Thermo Fisher) supplemented with 15% KSR (KO serum replacement, Thermo Fisher), 1 × l-glutamine (Thermo Fisher), 1 × Pen/Strep (Thermo Fisher), 1 × sodium pyruvate (Thermo Fisher), non-essential amino acids (Thermo Fisher), and 100 µM 2-mercaptoethanol (Thermo Fisher). Smad inhibitors 10 µM SB431542 (Tocris, Bristol, UK) + 0.1 µM LDN193189 (Stemgent, Cambridge, MS, USA) were added to the medium from day #0 to day #5 (see Supplemental Table 1). Three more small molecules 3 µM CHIR99021 (Tocris), 10 µM DAPT 10 µM (Tocris), and 10 µM SU5402 (Tocris) were added from day #2 to day #12. If overconfluent, cultures were split on day #2/day #3 to guarantee optimal differentiation [46]. From day #5 to day #12, the KSR medium was gradually transitioned to N2B27 medium (Neurobasal, Thermo Fisher) and added with 1% N_2_ supplement (Thermo Fisher), 1% B27 supplement (Thermo Fisher), 1 × glutamine (Thermo Fisher), and 1 × pen strep (Thermo Fisher). On day #11, young neurons were detached with TrypLE Express (Thermo Fisher) and replated on GFR (growth factor reduced) Matrigel (Corning)-coated coverslips. On day #13, the cultures were shifted to neural differentiation medium (Neurobasal, Thermo Fisher), supplemented with 1% N_2_ supplement (Thermo Fisher), 1% B27 (Thermo Fisher), 1% Pen-Strep (Thermo Fisher), 1% l-glutamine (Thermo Fisher), 25 ng/ml GDNF (Peprotech, London, UK), 25 ng/ml BDNF (Peprotech), 25 ng/ml NGF (Peprotech), 25 ng/ml NT3 (Peprotech), and 1 µM laminin (Sigma-Aldrich). CHIR99021 was added to neural differentiation medium for 3 days (from day #13 to day #15). 1 µM AraC (Sigma-Aldrich) was included in differentiation medium for 1 week (from day #13 to day #20) to kill off dividing cells. The treatment with AraC was repeated, if necessary, every other week (up to 3 cycles). Differentiation was usually extended from 2 to 4 weeks.

### Transfection and RNA interference

Transfection of HeLa cells was performed using Metafectene Pro from Biontex (Martinsried, Germany), as indicated by the manufacturer. After 20 h of transfection, cells were processed for DQ-BSA dequenching assay. Transfection of cells with siRNA was performed using Metafectene SI from Biontex (Martinsried, Germany) as indicated by the manufacturer. Cells were analyzed after 5 days of transfection. Small interfering RNAs (siRNAs) were purchased from MWG-Biotech (Ebersberg, Germany).

Rab7a siRNA efficiency in silencing was reported previously [36]: sense sequence 5′-GGAUGACCUCUAGGAAGAATT-3′ and antisense sequence 5′-UUCUUCCUAGAGGUCAUCCTT-3′. Control RNA was used as a negative control: sense sequence 5′-ACUUCGAGCGUGCAUGGCUTT-3′ and antisense sequence 5′-AGCCAUGCACGCUCGAAGUTT-3′.

### EGF internalization and EGFR degradation assays

For the EGF internalization assay, control and CMT2B skin fibroblasts were incubated overnight in starvation medium (0.5% BSA, 20 mm HEPES, pH 7.3, in DMEM). Cells were subsequently incubated for 1 h at 4 °C with 0.8 mg/ml rhodamine-labeled EGF (Thermo Fisher, E3481) in starvation medium and then washed several times with starvation medium. After incubation at 37 °C in complete DMEM medium for different time points (30 min, 1 h and 2 h), cells were fixed, mounted on slides, and processed for confocal microscopy. Zen 2011 software (Carl Zeiss, Oberkochen, Germany) was used for image capture and to calculate weighted colocalization coefficient of EGF and LAMP1. For EGFR degradation assay, control and CMT2B skin fibroblasts were incubated for 1 h at 37 °C with serum- and antibiotic-free DMEM and 10 μg/ml cycloheximide, then stimulated with EGF (50 ng/ml) for different times (15, 60, 180, 360 min) and lysed with RIPA buffer (50 mM Tris–HCl, pH 8.0, with 150 mM sodium chloride, 1.0% Igepal CA-630 (NP-40), 0.5% sodium deoxycholate, and 0.1% sodium dodecyl sulfate) plus protease inhibitor cocktail (Roche, Mannheim, Germany). The levels of degraded EGFR were determined by western blotting. When indicated, 100 μM chloroquine was added to the cells 3 h before harvesting.

### Analysis of cathepsin D synthesis

Cells were treated with 10 μg/ml cycloheximide (to inhibit protein synthesis) for 50 h, lysed with 2 × Laemmli buffer supplemented with DTT (dithiothreitol) and subjected to western blot analysis. Bands were quantified by densitometry using ImageJ software (National Institutes of Health).

### Cathepsin activity assays

Cathepsin D Activity Fluorometric Assay (K143-100, BioVision, Milpitas, CA, USA) utilizes the cathepsin-D substrate sequence GKPILFFRLK(Dnp)-D-R-NH2 labeled with fluorescent MCA (7-methoxycoumarin-4-acetic acid). 2 × 10^4^ control and CMT2B cells were collected and read in a fluorometer equipped with a 328-nm excitation filter and 460-nm emission filter. Cathepsin D activity was expressed by relative fluorescence units (RFU) per million cells and by RFU fold increase of CMT2B fibroblasts against control fibroblasts. Cathepsin B and Cathepsin L Activity Assay Kits (BioVision, K140-100 e K142-100) utilize, respectively, the preferred cathepsin-B RR and cathepsin-L FR substrate sequence labeled with fluorescent AFC (7-amino-4-trifluoromethylcoumarin). 2 × 10^4^ control and CMT2B fibroblasts were collected and read in a fluorometer equipped with a 400-nm excitation filter and 505-nm emission filter. Fold-increase in cathepsin-B or cathepsin-L activity was determined by comparing the relative fluorescence units (RFU) measured in CMT2B and control fibroblasts.

### DQ-BSA dequenching assay

Cells were grown on glass coverslips and treated with DQ-BSA. Fibroblasts were treated with DQ Red BSA (10 μg/ml, Thermo Fisher, D12051) for 48 h, neurons with DQ Green BSA (50 μg/ml, Thermo Fisher, D12050) for 24 h and HeLa cells with DQ Green BSA (10 μg/ml) for 6 h. Fluorescence was quantified with Zeiss LSM 700 confocal microscope.

### Western blotting

Control and CMT2B fibroblasts and neurons were lysed with Laemmli buffer [100 mM Tris–HCl, pH 6.8, 4% (w/v) SDS, 0.2% (w/v) bromophenol blue, 20% glycerol and 200 mM DTT (dithiothreitol)]. Lysates were loaded on SDS-PAGE and separated proteins were transferred onto PVDF membrane (Merck Millipore). The filter was blocked in 5% milk in PBS for 30 min at room temperature, incubated with the appropriate primary antibody and then with a secondary antibody conjugated with HRP (diluted 1:5000). When phosphorylation was monitored, cells were lysed in the presence of phosphatase inhibitors (PhosSTOP, Roche). Bands were visualized using Western blot Luminol Reagent (Santa Cruz) or Western Bright ECL kit (Advansta, Menlo Park, CA, USA) or ClarityMax (Bio-Rad). The signal was captured on a film, avoiding saturation, to ideally compare samples in the linear range of detection. To prevent saturation of the signal, we previously estimated proper loading amounts. To reduce variability, we did not quantify signals that decayed too quickly and we avoided comparing very weak bands that may be outside the linear range. Films were then scanned at 600 dpi resolution and quantified using ImageJ (National Institutes of Health). Each band was quantified selecting rectangular areas and subtracting the film background. Bands relative to the protein of interest and to the related tubulin loading control were quantified. Measurement of each protein was then normalized on the related tubulin loading control and CMT2B normalized measurements were compared to control normalized measurements obtained from the same gel.

### Confocal immunofluorescence microscopy

Fibroblasts grown on coverslips were permeabilized, fixed and incubated with primary and secondary antibodies as described previously [47] and viewed with Zeiss LSM 700 confocal microscope. HeLa cells were fixed for 20 min in 3% paraformaldehyde, permeabilized with 0.1% TX100 in PBS and then incubated with primary and secondary antibodies diluted in 0.1% saponin in PBS. Then, washing, cells were stained with DAPI and coverslips were mounted and viewed with Zeiss LSM 700 confocal microscope.

iPS cells or peripheral neurons were fixed for 20 min in 4% paraformaldehyde, blocked and permeabilized for 20 min with 10% NGS, 1% BSA, and 0.1% TX100. Alternatively, cells were fixed and permeabilized for 10 min in ice‐cold methanol at − 20 °C. After incubation with primary antibody overnight at 4 °C, the coverslips were washed, incubated with the secondary antibody for 30 min, washed, and mounted with DAPI (H-1200, Vector Laboratories, Burlingame, CA, USA) and viewed with Leica SP5 confocal microscope.

### Electron microscopy

Cells were fixed in 2% glutaraldehyde in 0.2 M Hepes, pH 7.4, at room temperature for 2 h. During fixation, the cells were scraped off the culture dish and pelleted. After postfixation in 1% osmium tetroxide, cells were dehydrated in ethanol and embedded in epoxy resin. Sections were cut with a diamond knife and contrasted with uranyl acetate and lead citrate. Microscopy was performed with a Jeol JEM 1400 Plus transmission electron microscope.

### Real-time PCR

For RT-PCR on fibroblasts, RNA was isolated from cells using an RNeasy Micro kit according to the manufacturer’s instructions (Qiagen, Hilden, Germany). For RNA retrotranscription protocol, we used SuperScript II Reverse Transcriptase (Invitrogen) according to the manufacturer’s instructions. Briefly, a mixture (12 µl) containing 4 µg of RNA, 10 mM of deoxynucleotides and 40 ng of random primers (Promega, Madison, WI, USA) was heated at 65 °C for 5 min. First-strand cDNA synthesis was carried out with SuperScript II Reverse Transcriptase in the presence of dithiothreitol (0.01 M) and ribonuclease inhibitor (40 U, RNaseOUT, Invitrogen) at 42 °C for 50 min. Reactions were stopped by heat inactivation at 70 °C for 15 min.

Quantitative real-time PCR was carried out with Power SYBR Green (Applied Biosystems, Foster City, USA) using Applied Biosystems 7900HT Fast Real-time PCR System.

The primers used were:

GAPDH: *forward*: 5′-GGTGGTCTCCTCTGACTTCAACA-3′,

*reverse*: 5′-GTTGCTGTAGCCAAATTCGTTGT-3′.

Cathepsin B: *forward:* 5′-CTGTCGGATGAGCTGGTCAAC-3′,

*reverse*: 5′-TCGGTAAACATAACTCTCTGGGG-3′.

Cathepsin D: *forward*: 5′-CAGAAGCTGGTGGACCAGAAC-3′,

*reverse*: 5′-TGCGGGTGACATTCAGGTAG-3′.

Cathepsin L: *forward*: 5′-GCTAATGACACCGGCTTTGT-3′,

*reverse*: 5′-TTTCAAATCCGTAGCCAACC-3′.

LAMP1: *forward:* 5′-ACGTTACAGCGTCCAGCTCAT-3′,

*reverse*: 5′-TCTTTGGAGCTCGCATTGG-3′.

LAMP2: *forward*: 5′-TGCTGGCTACCATGGGGCTG-3′,

*reverse*: 5′-GCAGCTGCCTGTGGAGTGAGT-3′.

These were purchased from Eurofins Genomics (Ebersberg, Germany).

The thermal profile used for Real-time PCR was as follows: 1 cycle of 2 min at 50 °C; 1 cycle of 10 min at 95 °C; 40 cycles of 15 s at 95 °C, 1 min at 55 °C; 1 cycle of 15 s at 95 °C and 15 s at 60 °C. The specificity of PCR products was checked by performing a melting-curve test. The relative expression level was calculated using the comparative *C*_T_ method and expressed as a “fold change”. The quantitative values were obtained from the mean minimal cycle threshold (*C*_T_) calculated from triplicate reactions. The fold change, measured as the amount of target gene normalized to the endogenous reference gene GAPDH, was given by 2^−^^∆^^∆C^_T_, where ∆*C* = *C*_Ttarget_ − *C*_TGAPDH_ and ∆∆CT = ∆_CTsample_ − ∆C_control_. Negative fold change was calculated using the formula − 1/2^−^^∆∆^^*C*^_T_. The relative quantification was considered significant when there was a minimum of twofold change.

For RT-PCR on iPS or iPS-derived cells, in brief, iPS cell clones and differentiated iPSCs tested for pluripotency (Trilineage differentiation Kit, Stem Cell Technologies) were homogenized with TRIzol reagent (15596026; Invitrogen) and total RNA was extracted with chloroform and precipitated with isopropanol. A portion (500 ng) of total RNA was reverse transcribed using High-Capacity cDNA Reverse Transcription Kits (4368814, Applied Biosystems), according to the manufacturer’s instructions. Semiquantitative RT-PCR analyses were performed with GoTaq G2 Flexi DNA Polymerase (M7805, Promega). Primer sequences are shown in Supplemental Table 2, while primers used to test iPSCs for Sendai virus clearance derived from CytoTune2.0‐iPS Sendai Reprogramming Kit (Thermo Fisher).

### Wound-healing assay

Confluent monolayer of fibroblasts was wounded with a pipette tip. The cell debris was washed out with PBS. Cells were imaged at the moment of the scratch (T0) and after 15 h using a 10 × objective of EVOS digital microscope. Accumulated distance was measured using photoshop software as the total distance that cells traveled in a certain amount of time.

### Gelatin zymography

The conditioned medium was collected from cells cultured for 24 h in serum-free medium, the sample was concentrated using Amicon^®^ Ultra (Merck Millipore) and quantified by Bradford assay. Each sample was adjusted to 15 µg/ml, and 10 µl was loaded in non-reduced protein sample buffer on 7.5% SDS-PAGE gel containing 1 mg/ml gelatin (Biorad). The gel was washed in a specific buffer (2.5% Triton X-100, 50 mM Tris–HCl pH 7.5, 5 mM CaCl_2_, 1 µM ZnCl_2_) to remove SDS and then three times in distilled water. After washing, the gel was incubated at 37 °C for 24 h in a buffer containing 1% Triton X-100, 50 mM Tris–HCl pH 7.5, 5 mM CaCl_2_, 1 µM ZnCl_2_ and then was stained with 0.5% Coomassie Blue R-250 (Sigma-Aldrich) and destained in 40% methanol and 10% acetic acid.

### Rac1 activity assay

Rac1 activity was tested following the manufacturer’s protocol with Rac1 Pull-down Activation Assay Biochem Kit (BK035, Cytoskeleton). Briefly, lysates were mixed with GST-Pak-PBD beads for 1 h at 4 °C. After washing, the pelleted beads were resuspended in Laemmli sample buffer and subjected to SDS–polyacrylamide gel electrophoresis. GTP-bound Rac1 was detected by western blot analysis using anti-Rac1 antibody. The total amount of Rac1 was detected by immunoblotting of the whole cell lysates.

### Teratoma formation

iPSCs were preincubated with mTeSR1 (Stem Cell Technologies), added with ROCK inhibitor (Stem Cell Technologies) for 1 h, harvested by Accumax (Sigma-Aldrich) treatment, collected into tubes, and centrifuged, and the pellets were suspended in PBS 1 × (Sigma-Aldrich) with ROCK inhibitor. One-third of the cells from a confluent 35 mm dish was injected in the testis of 8 weeks old Fox Chase SCID Beige mice (Strain Code: 250, Charles River). Six weeks after injection, tumors were dissected and fixed with formaldehyde solution. Paraffin-embedded tissue was sliced and stained with hematoxylin and eosin. All animal experiments were approved and performed in compliance with the guidelines of San Raffaele Institutional Animal Care and Use Committee.

### DNA sequence analysis of RAB7

Genomic DNA was extracted from pelleted neurons using GenElute Mammalian Genomic DNA Miniprep Kit (Sigma-Aldrich) according to the manufacturer’s protocol. 100 ng of genomic DNA was amplified by PCR using the following primers for exon 4: 5′ CTGTGTCCTCACCTGTACTACC 3′ and 5′ GAAAAGAGTGGGTTAGGGAAGAAG 3′. PCR conditions for this reaction are: 94 °C for 7 min, (94 °C for 1 min, 55 °C for 30 s, 72 °C for 45 s) × 30 cycles followed by a final step at 72 °C for 7 min. PCR products were loaded on agarose gel and extracted using QIAEX II Gel Extraction Kit (Qiagen, Hilden, Germany). Samples were shipped to Eurofins Genomics (Ebersberg, Germany) for sequencing.

### Neurite length analysis

Neurite length was measured by IncuCyte^®^ S3 Live-Cell Analysis System (Essen BioSCience). Briefly, young neurons were plated in 24-well plate (20,000 cells/well). Bright field (contrast) real-time automated acquisition and measurements for live cells were performed every 4 h for the first six consecutively days after neuron plating. Two wells per clones were plated and sixteen field per well were analyzed (objective 20×). Neurite length was measured as detected by the IncuCyte Zoom software as the total combined length of all neurites (mm) detected per mm^2^. All data are expressed relative to the respective day 0 of maturation. Each group is represented by mean ± SEM (*n* = 32).

### Statistical analysis

Data were statistically analyzed using Student's *t* test and Mann–Whitney *U*- test (GraphPad Prism4 software) (*p < 0.05, **p < 0.01 and *p* < 0.001). Error bars represent SEM. Experiments were performed at least in triplicate.

## Results

### Late endocytic protein expression is altered in CMT2B fibroblasts

To investigate possible endocytic and lysosomal defects in CMT2B, we used previously isolated dermal fibroblasts from a CMT2B patient carrying the RAB7^V162M^ mutation and from a healthy age- and sex-matched control [43].

**Fig. 1:**
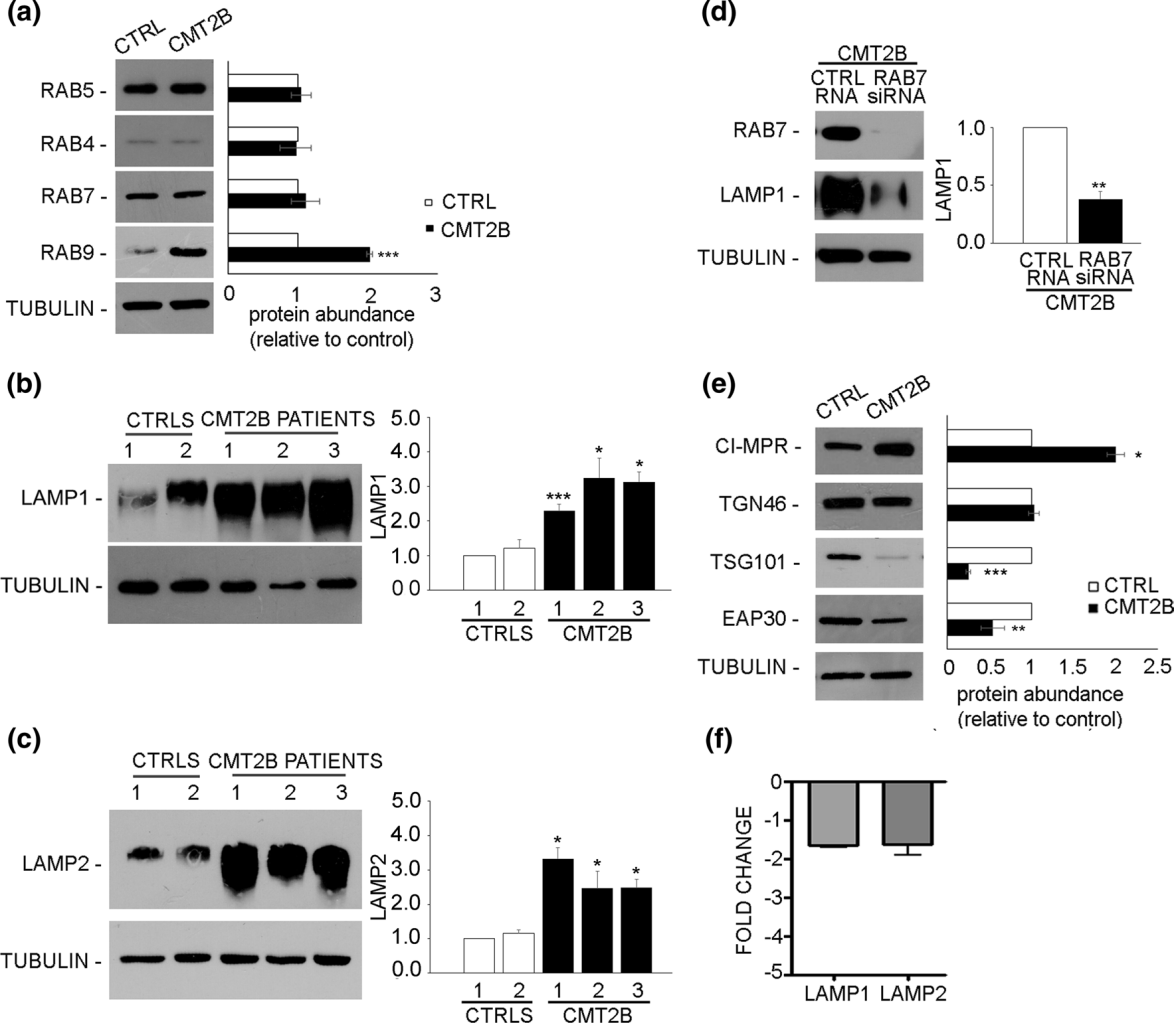
Analysis of endosomal and lysosomal markers in control and CMT2B fibroblasts. **a** Lysates of control and CMT2B patient 1-derived skin fibroblasts carrying the RAB7^V162M^ mutation were analyzed by immunoblotting using anti-RAB5, anti-RAB4**,** anti-RAB7 and anti-RAB9. **b**, **c** Lysates of two controls and three CMT2B skin fibroblasts carrying the RAB7^V162M^ mutation were analyzed by immunoblotting using anti-LAMP1 (**b**) and anti-LAMP2 (**c**) antibodies. **d** CMT2B fibroblasts were silenced for RAB7 and then LAMP1 abundance was analyzed by immunoblotting using anti-LAMP1 antibody. **e** Lysates of control and CMT2B patient 1-derived skin fibroblasts were analyzed by immunoblotting using anti-CI-MPR, anti-TGN46, anti-TSG101 and anti-EAP30 antibodies. Bands were quantified using NIH ImageJ and normalized against tubulin. **f** The amount of LAMP1 and LAMP2 transcripts was quantified, compared to the GAPDH transcript as control, using real-time PCR in control and CMT2B patients-derived fibroblasts. All data represent the mean ± SEM of at least three experiments. Statistical analysis was performed using Student’s *t* test with control fibroblasts as referring sample. *p < 0.05; **p < 0.01; ***p < 0.001

We analyzed first the expression of early endocytic RABs and found comparable amounts of RAB5, a protein controlling early endosomal homotypic fusion and transport from plasma membrane to early endosome [47, 48], as well as of RAB4, a protein regulating different steps of endocytic recycling [49] (Fig. 1a). Next, we looked at late endocytic RAB proteins, such as RAB7 and RAB9, whose abundance increases during early to late endosome transition [1, 50]. Similar amounts of RAB7, which is mutated in CMT2B neuropathy, were found in CMT2B compared to control fibroblasts (Fig. 1a). However, we observed a strong increase in the levels of RAB9 (Fig. 1a) of about twofold in CMT2B compared to control cells. Subsequently, we investigated the abundance of other late endosomal and lysosomal markers such as lysosomal associated membrane protein 1 and 2 (LAMP1 and LAMP2) [1, 51] in fibroblasts derived from three CMT2B patients of the same Italian family [33] and from two healthy individuals. We observed that, compared to the controls, all CMT2B patients showed higher abundance of LAMP1 and LAMP2, although to a different extent (Fig. 1b, c). We then silenced RAB7 in CMT2B fibroblasts and observed reduced abundance of LAMP1, thus suggesting that the different expression of LAMP proteins in CMT2B cells is most likely related to RAB7 (Fig. 1d).

We then looked at the expression of cation independent mannose-6-phopshate receptor (CI-MPR), a protein involved in transport between Golgi and endosomes, and TGN46, localized to the trans-Golgi network [51–53]. We found that, in CMT2B fibroblasts, the expression of CI-MPR was twofold higher than in control cells (Fig. 1e), while the expression of TGN46 did not change (Fig. 1e).

Sorting events play a crucial role in endocytosis and control correct cargo shipment to lysosomes. Among proteins important for sorting at the level of early endosomes, endosomal sorting complex required for transport (ESCRT) proteins are fundamental for targeting signaling receptors to degradation [54]. Therefore, we decided to investigate the abundance of some of these proteins in CMT2B cells. We found that tumor susceptibility gene 101 (TSG101) was strongly decreased in CMT2B fibroblasts compared to control cells (Fig. 1e), while the expression of ELL (eleven-nineteen lysine-rich leukemia) associated protein 30 (EAP30), the homolog of yeast VPS22, was about 40% lower in CMT2B fibroblasts (Fig. 1e), suggesting that ESCRT proteins are down-regulated in CMT2B.

To establish whether the observed changes in the amount of late endosomal and lysosomal markers were a consequence of mRNA abundance, we measured LAMP1 and LAMP2 mRNA expression using real-time PCR (Fig. 1f). As the relative quantification is considered significant when there is minimum of twofold change, the mRNA levels of LAMP1 and LAMP2 were not significantly changed (Fig. 1f).

Altogether, these results indicate that in CMT2B fibroblasts, the abundance of early endocytic markers is not affected, the expression of sorting proteins regulating the lysosomal biogenesis is reduced, while the expression of lysosomal protein is increased, suggesting that the late endocytic pathway is altered and that these changes are due to modified RAB7 activity.

### CMT2B cells have more lysosomes

**Fig. 2:**
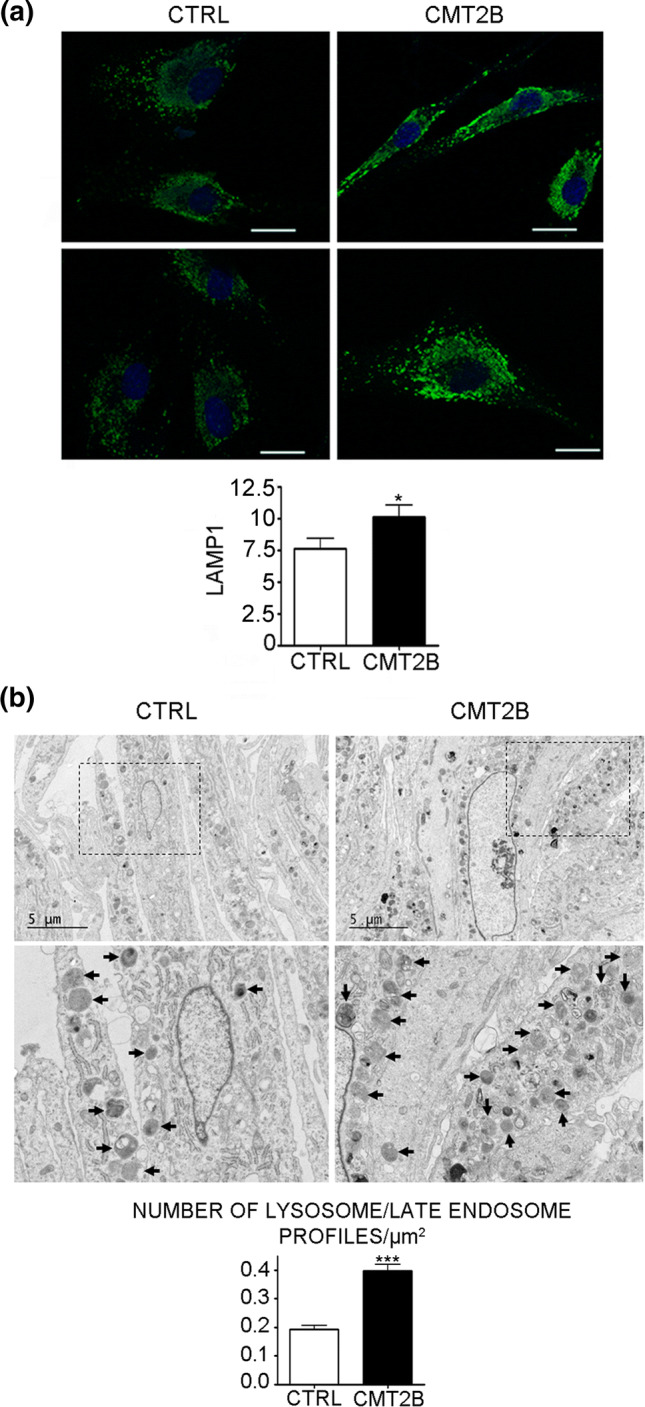
Electron microscopy of control and CMT2B patient 1 fibroblasts. **a** Control and CMT2B fibroblasts from patient 1 carrying the RAB7^V162M^ mutation were fixed and immunolabeled with anti-LAMP1 followed by Alexa488-conjugated secondary antibody, while nuclei were stained with DAPI. Bars 20 µm. LAMP1 intensities of at least 50 cells per sample were measured using ImageJ. Graphs were generated with GraphPad. Mann–Whitney *U* test was used for statistical analysis and control fibroblasts were selected as referring sample. *p < 0.05. **b** The boxed areas are shown at higher magnification in the lower panels. Arrows indicate some of the lysosomes. Bars 5 µm. Graphs of lysosome number quantification were generated with GraphPad. Mann–Whitney *U* test used for statistical analysis and control fibroblasts were selected as referring sample. ***p < 0.001

We next investigated the abundance of lysosomes in CMT2B cells by immunofluorescence analysis. In control cells, using antibodies against LAMP1, a marker of late endosome and lysosomes, we observed, as expected the presence of many LAMP1-positive organelles mostly concentrated around the nucleus (Fig. 2a). Staining of CMT2B cells revealed a similar distribution, but a stronger staining of these structures present in the perinuclear area (Fig. 2a), as confirmed by quantification (Fig. 2a).

**Fig. 3:**
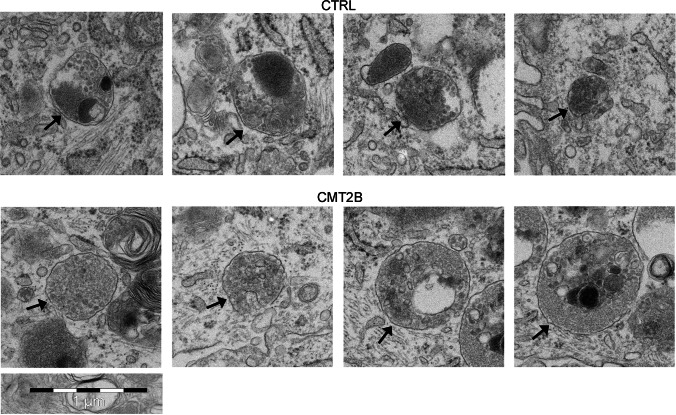
Electron microscopy of multivesicular bodies from control and CMT2B patient 1 fibroblasts. Arrows indicate multivesicular bodies (MVBs) observed in control and CMT2B patient 1 carrying the RAB7^V162M^ mutation. Bar 1 µm

Immunoblotting (Fig. 1b, c) and immunofluorescence data (Fig. 2a) suggest that the amount of lysosomes may be increased in patient cells. To investigate this, we performed electron microscopy, demonstrating that patient cells were actually filled with lysosomes (Fig. 2b). Quantification of the number of lysosome profiles in thin sections demonstrated that CMT2B cells have about twice as many lysosomes as compared to control cells (Fig. 2b), while no alterations in the formation of multivesicular bodies were observed (Fig. 3).

Altogether, these data indicate that CMT2B cells have more lysosomes.

### CMT2B fibroblasts show higher lysosomal activity

**Fig. 4:**
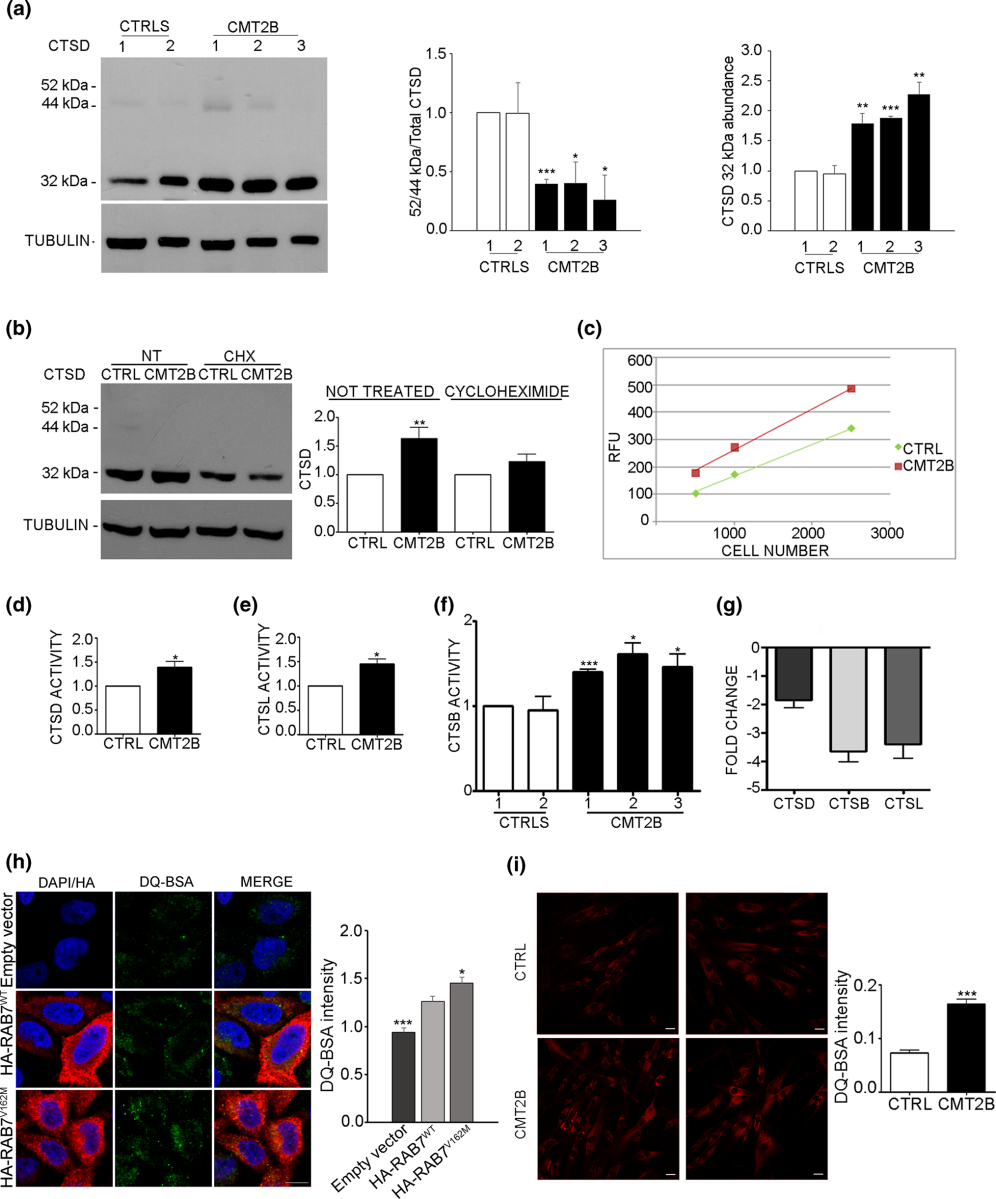
Lysosomal activity in CMT2B fibroblasts carrying the RAB7^V162M^ mutation. **a** Lysates of two controls and CMT2B fibroblasts from three patients were subjected to immunoblotting using anti cathepsin D (CTSD) and anti-tubulin antibodies. Intensities of bands were measured by densitometry and normalized against tubulin. **b** Lysates of control and CMT2B fibroblasts from patient 1 untreated or treated with cycloheximide for 50 h were subjected to immunoblotting using anti cathepsin D and anti-tubulin antibodies. Intensities of bands were measured by densitometry and normalized against tubulin. **c** Different dilutions of control and CMT2B patient 1 lysates were tested for ability to cleave the MCA-labeled synthetic substrate. RFU per each dilution was evaluated after fluorimetric analysis. **d** Quantification of fluorescence data to evaluate cathepsin-D activity in control and CMT2B fibroblasts (patient 1). **e** The ability of cathepsin-L (CTSL) to cleave the substrate was evaluated in both control and CMT2B patient 1-derived fibroblasts. **f** Fibroblasts derived from two distinct control individuals and three CMT2B patients were analyzed to test cathepsin B (CTSB) activity. **g** The amount of cathepsin-D, L and B transcripts was quantified, compared to the GAPDH transcript as control, using real-time PCR, in control and CMT2B patients-derived fibroblasts. **h** HeLa cells were transfected for 24 h with the empty vector or plasmids encoding HA-tagged RAB7^WT^ or CMT2B-causing mutant RAB7^V162M^. Cells were incubated for 6 h with DQ Green BSA and then processed for immunofluorescence analysis. Bar 10 µm. DQ BSA intensities were measured using ImageJ and Corrected Total Cell Fluorescence (CTCF) was calculated. Data represent the mean ± SEM of at least 50 cells analyzed. Student’s *t* test was used for statistical analysis. Statistical comparisons of the samples are with RAB7^WT^. *p < 0.05; ***p < 0.001. **i** Control and CMT2B fibroblasts from patient 1 were treated with DQ-BSA for 48 h, then fixed and observed on a confocal microscope. Bars 20 µm. The intensities of manually determined areas for each single cell were measured using Image J. At least 50 cells per sample were analyzed. Graphs were generated with GraphPad. All data represent the mean ± SEM of at least three independent experiments. Student’s *t* test or Mann–Whitney *U *test (for DQ-BSA quantification) was used for statistical analysis and control fibroblasts were selected as referring sample. *p < 0.05; **p < 0.01, ***p < 0.001

The higher abundance of late endocytic proteins and of lysosomes in CMT2B cells prompted us to hypothesize a higher lysosomal activity in CMT2B fibroblasts. Cathepsin-D is a lysosomal enzyme synthesized as an inactive precursor (pre-pro-cathepsin), converted into pro-cathepsin D (52 kDa) in the endoplasmic reticulum and further processed into late endosomes and lysosomes into the 44-kDa form and then into the 32-kDa mature form [55–57]. Defects in cathepsin-D maturation correlate with alterations of lysosomal functions. Using a specific antibody able to detect the different immature and mature forms of cathepsin D, we could detect the immature forms both in control and CMT2B fibroblasts (Fig. 4a). Quantification of the ratio of immature cathepsin D on total cathepsin D indicates that cathepsin D processing is more efficient in CMT2B cells (Fig. 4a). In addition, quantitative analysis of the total amount of mature cathepsin D revealed that cathepsin D abundance in CMT2B fibroblasts was about twofold higher than in control cells (Fig. 4a). Treatment with cycloheximide, an inhibitor of protein synthesis, lowered this increase, suggesting that the higher amount of cathepsin D is due to increased protein synthesis (Fig. 4b).

Cathepsin D is important not only for lysosomal activity, but also for lysosome-mediated apoptosis [55]. T understand whether cathepsin D increase in CMT2B cells correlates with higher degradation activity, we performed a cathepsin D activity assay based on fluorescence release upon cleavage of a substrate. CMT2B fibroblasts showed higher fluorescence (Fig. 4c) and quantification revealed that cathepsin D activity in CMT2B cells was approximately 40% higher than in control fibroblasts (Fig. 4d).

To confirm increased lysosomal activity, we decided to test the enzymatic activity of other lysosomal proteins such as cathepsin-L and cathepsin-B using similar fluorescence-based assays on patient 1 fibroblasts. Both cathepsin L and cathepsin B activities were about 40% higher in CMT2B cells compared to control cells (Fig. 4e, f), thus confirming increased lysosomal activity. To establish whether the higher activity of cathepsins observed was a consequence of alterations in gene expression, we analyzed mRNA abundance and measured cathepsin B, D and L mRNA expression using real-time PCR (Fig. 4f). The mRNA levels of cathepsin D were not significantly changed, while cathepsin B and L mRNAs were decreased, possibly due to a compensatory mechanism to counteract the higher amount and activity of these proteins (Fig. 4g).

To further investigate lysosomal activity, we used the DQ™ BSA assay in which cells are incubated with the self-quenched DQ™ BSA, whose cleavage by proteases in an acidic compartment generates a highly fluorescent product. HeLa cells transfected with the empty vector or with a construct encoding HA-tagged RAB7^WT^ or the CMT2B-causing mutant RAB7^V162M^ were incubated with DQ-BSA. After 6 h of incubation with DQ™ Green BSA, HeLa cells expressing CMT2B-causing mutant RAB7^V162M^ showed higher fluorescence compared to cells transfected with empty vector and plasmid encoding HA-tagged RAB7^WT^ (Fig. 4h). Similarly, control fibroblasts and CMT2B fibroblasts from patient 1 were analyzed after 48 h of incubation with DQ™ Red BSA. Control fibroblasts displayed a lower level of fluorescence compared to CMT2B fibroblasts and quantification revealed an approximately twofold increase of fluorescence in CMT2B compared to control cells (*p* < 0.0001) (Fig. 4i).

Altogether, these data demonstrate that lysosomal activity in CMT2B cells is higher than in control cells and that this is caused by the expression of the RAB7^V162M^ mutant protein.

### EGFR degradation is higher in CMT2B cells

The higher expression of late endocytic proteins, the lower abundance of sorting regulators and the increased number of lysosomes and lysosomal activity suggest alterations of the degradative pathway in CMT2B fibroblasts. RAB7 regulates trafficking and degradation of EGFR and previous data on EGFR degradation obtained on cells transiently or stably transfected with the CMT2B-causing RAB7 mutant proteins produced conflicting results. In fact, transient expression of CMT2B-causing RAB7 mutant proteins in HeLa cells caused normal or increased EGFR degradation [35, 36], while in Hela and PC12 stable cell lines expressing these mutant proteins inhibition of EGFR degradation was reported [58].

**Fig. 5:**
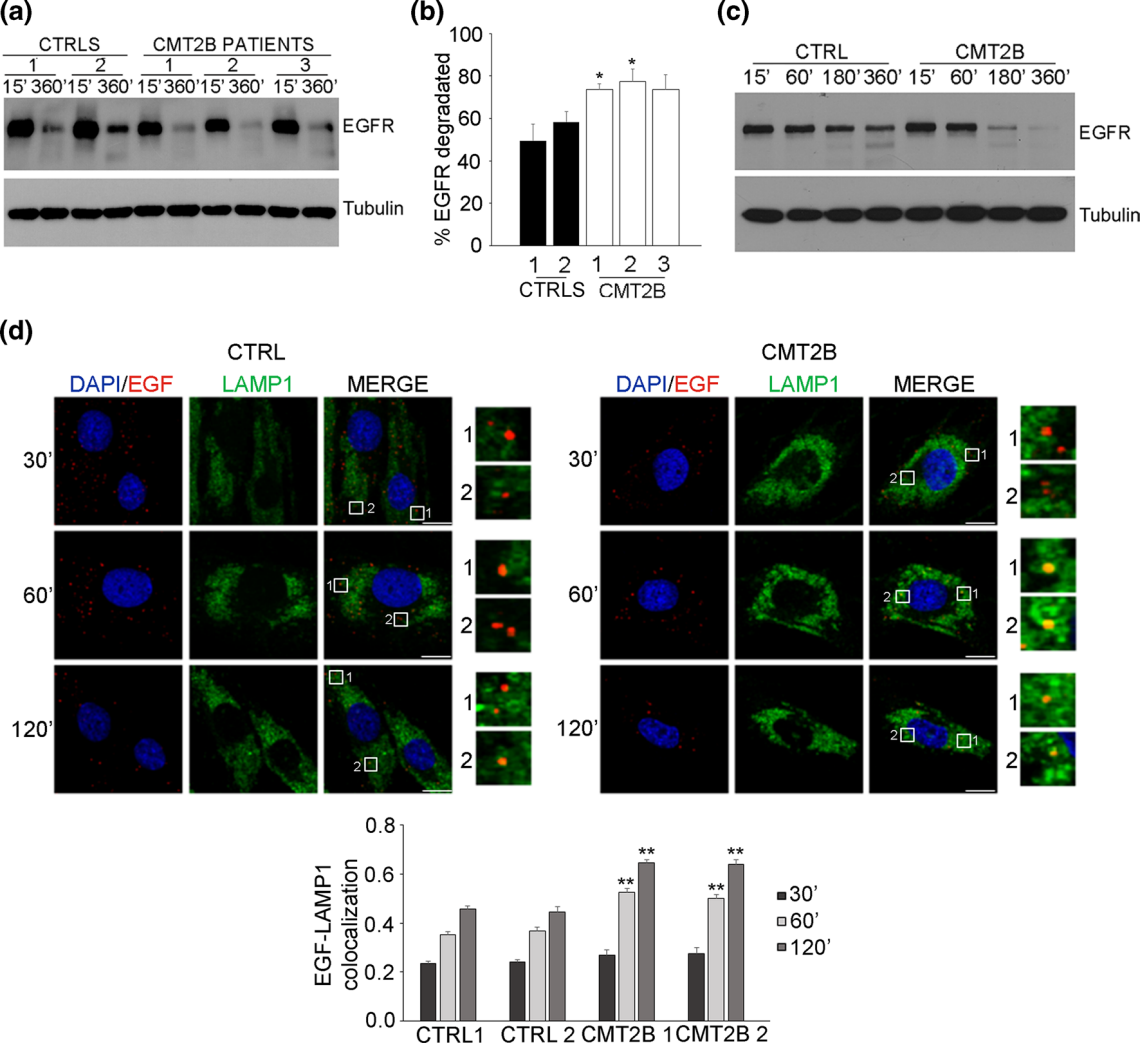
Degradation activity is higher in CMT2B fibroblasts carrying the RAB7^V162M^ mutation. **a** Fibroblasts derived from two healthy individuals and three CMT2B patients were incubated with cycloheximide for 1 h and subsequently stimulated with EGF for 15 and 360 min. Cell lysates were analyzed by immunoblotting with antibody against EGFR and tubulin. **b** Densitometric analysis was performed with NIH ImageJ normalizing against tubulin. Statistical analysis was performed using Student’s *t* test with control fibroblasts as referring sample. **c** Fibroblasts derived from control and CMT2B (patient 1) were incubated with cycloheximide for 1 h and subsequently stimulated with EGF for the indicated times. Cell lysates were analyzed by immunoblotting with antibody against EGFR and tubulin. **d** Skin fibroblasts derived from two healthy individuals and two CMT2B patients (patients 1 and 2) were incubated overnight in starvation medium and then incubated for 1 h at 4 °C with rhodamine-EGF. After several washing, cells were incubated in complete DMEM medium at 37 °C for 30 min, 1 h and 2 h and then fixed, permeabilized, immunolabeled with anti-LAMP1 followed by Alexa488-conjugated secondary antibody while nuclei were stained with DAPI. For each image, magnifications of the boxed areas are shown. Bars 10 µM. Data represent the mean ± SEM of at least 50 cells of three independent experiments. Statistical analyses were performed using Student’s *t* test with control fibroblasts as referring sample. **p < 0.01

To solve this issue and establish if also EGFR degradation is affected in CMT2B cells, we measured EGFR degradation in CMT2B fibroblasts carrying the RAB7^V162M^ mutation (Fig. 5a). Control and CMT2B cells were able to degrade EGFR efficiently but, notably, CMT2B fibroblasts showed a higher degradation activity at 360 min. In fact, quantification of EGFR degradation at 360 min in CMT2B patient 1 and patient 2 was significantly higher than controls, while in patient 3 was higher but not statistically significant (Fig. 5b). EGFR degradation assay at different time points on control and patient 1 fibroblasts revealed increased degradation both at time point 180 and 360 min (Fig. 5c). Our results are in agreement with previous data obtained on transiently transfected HeLa cells, but also with data on transferrin receptor degradation that was increased in fibroblasts from CMT2B patient 1 [35, 36, 43].

To evaluate if higher degradation is due to increased trafficking speed, we performed an EGF internalization assay and measured colocalization between internalized Rhodamine-EGF and LAMP1 at different time points. Interestingly, we found that in patients’ fibroblasts rhodamine-EGF and LAMP1 colocalization is higher at 1 h and 2 h compared to controls indicating that trafficking to late endosomes and lysosomes is faster in CMT2B fibroblasts compared to control cells (Fig. 5d).

These results, together with quantitative analysis of late endocytic proteins, strongly indicate that the endocytic degradative pathway is increased in CMT2B fibroblasts.

### EGFR signaling and cell migration are altered in CMT2B fibroblasts

**Fig. 6:**
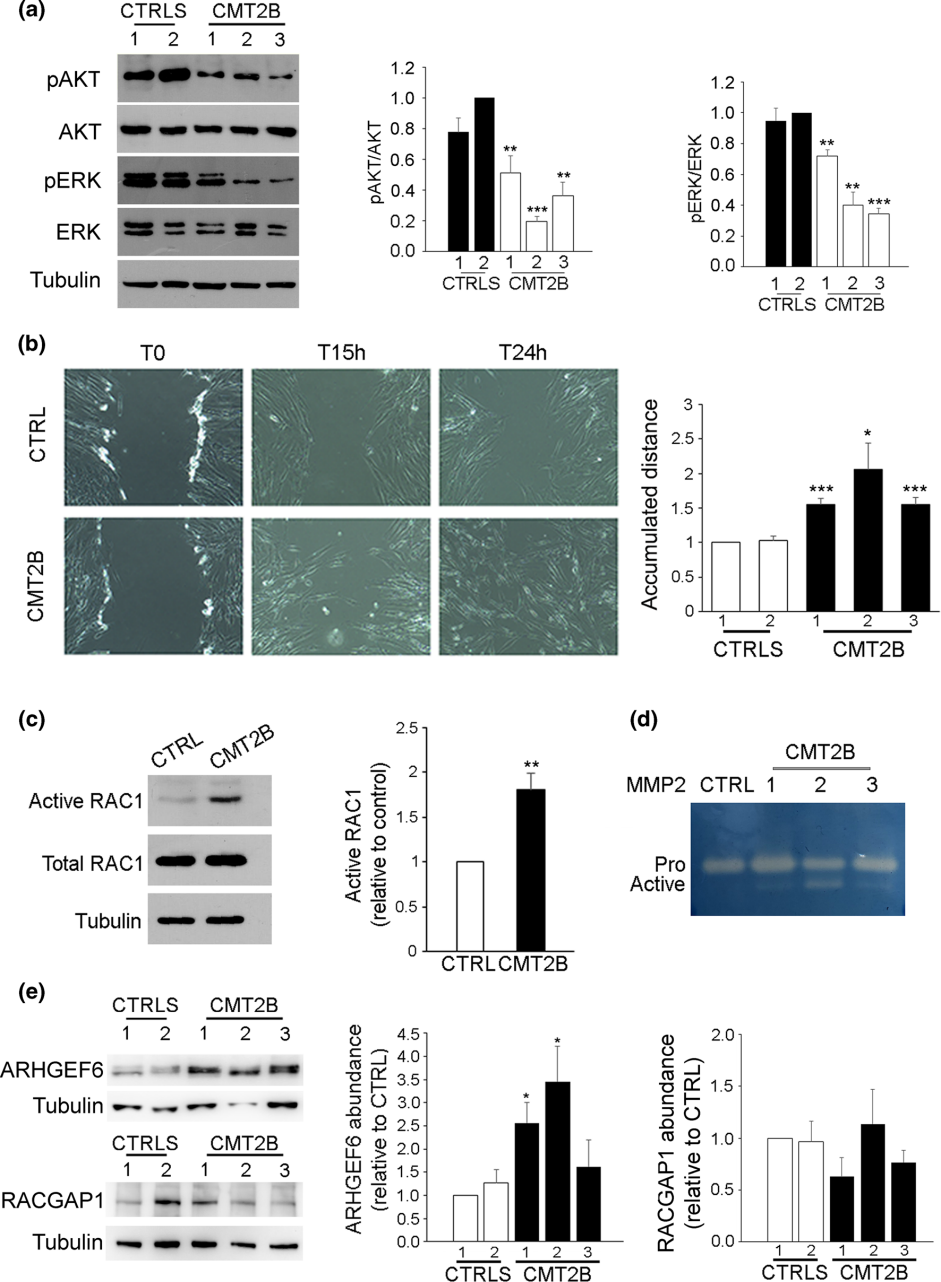
Rac1 activity and cell migration are altered in CMT2B fibroblasts carrying the RAB7^V162M^ mutation. **a** Lysates of two controls and three CMT2B fibroblasts were analyzed by immunoblotting using anti-AKT, anti-pAKT, anti-ERK and anti-pERK. Bands were quantified using NIH ImageJ and normalized against total protein. All data represent the mean ± SEM of at least three experiments. Statistical analysis was performed using Student’s *t* test with control fibroblasts as referring sample. *p < 0.05, *p < 0.01, ***p < 0.001. **b** Fibroblasts from control and CMT2B patients were imaged during wound healing assay. Images of initial time point (T0), 15 h and 24 h after the scratch are shown. Accumulated distance, the total distance that the cell traveled in a certain amount of time, is shown. Data represent the mean ± SEM of four experiments. Statistical analysis was performed using Student’s *t* test with control fibroblasts as referring sample. *p < 0.05; ***p < 0.001. **c** Lysates of control and CMT2B fibroblasts (patient 1) were subjected to RAC1 activation assay and then subjected to western blot analysis using anti-RAC1 and anti-tubulin antibody. Quantification of active RAC1 in control and CMT2B cells is shown. Data represent the mean ± SEM of at least three experiments. **p < 0.01. **d** Gelatin zymography was performed using conditioned medium of fibroblasts derived from a healthy individual and three CMT2B patients. Representative results are shown. **e** Lysates of two controls and three CMT2B skin fibroblasts were analyzed by immunoblotting using anti-ARHGEF6 and anti-RACGAP1 antibodies. Bands were quantified using NIH ImageJ and normalized against tubulin. All data represent the mean ± SEM of at least three experiments. Statistical analysis was performed using Student’s *t* test with control fibroblasts as referring sample. *p < 0.05

The activation of EGFR stimulates a number of signaling pathways, among which are phosphatidylinositol 3 kinase (PI3K/AKT) and Ras-mitogen-activated protein kinase (MAPK) pathways, to promote cell survival and proliferation [59]. To identify downstream factors of EGFR affected in CMT2B, we analyzed the abundance and activation of AKT and ERK proteins in fibroblasts derived from three CMT2B patients and from two healthy individuals. Consistently with increased degradation of EGFR, we observed in CMT2B fibroblasts from the three patients a significant reduction in the activation of both AKT and ERK (Fig. 6a).

EGFR is also important for the regulation of cell motility [19, 49]. As we demonstrated that the RAB7^V162M^ mutation affects EGFR degradation and, as a consequence, inhibits AKT and ERK signaling, we hypothesized inhibition of cell migration. To measure cell migration, we performed a wound-healing assay. Control and CMT2B fibroblasts were grown to confluence, the cell layer was scratched and the wound area was monitored at different time intervals. Interestingly, we observed an approximately twofold increase in cell migration for CMT2B fibroblasts compared to control cells at 15 h after the scratch (Fig. 6b). Given this unexpected result, we investigated other players in cell migration.

RAC1, a Ras-related small GTPase involved in several cellular pathways, is a member of the RHO family that regulates actin cytoskeleton during cell motility [60]. In addition, during cell migration, RAC1 activity is regulated by RAB7 [61]. Therefore, we analyzed the abundance and activation of RAC1 in CMT2B fibroblasts. We did not detect any differences in RAC1 protein amount between the control and CMT2B cells (Fig. 6c). However, we observed a significant increase of GTP-bound active RAC1 in CMT2B cells compared to controls, demonstrating that in these cells RAC1 is more activated (Fig. 6c).

Matrix metalloproteinases (MMPs) comprise a family of endopeptidases that degrade extracellular proteins promoting cell migration [62] and RAC1 is a mediator of MMP-2 activation [63]. Therefore, we monitored MMPs activity by gelatin zymography in control and CMT2B fibroblasts. In the medium of CMT2B cells, we detected two bands with different intensity corresponding to inactive and active MMP-2 (Fig. 6d) while in control cells only the inactive band was present.

To investigate the mechanism leading to increased RAC1 activation in CMT2B patients, we evaluated the expression of ARHGEF6 and RACGAP1 [64, 65]. We did not observe any difference in RACGAP1 expression between control and CMT2B fibroblasts, while we found an increased expression of ARHGEF6 in CMT2B fibroblasts (Fig. 6e) that could explain the increased RAC1 activation in these cells.

Altogether, these data demonstrate that EGFR signaling and cell migration are affected in CMT2B fibroblasts.

### CMT2B sensory neurons show higher lysosomal activity

**Fig. 7:**
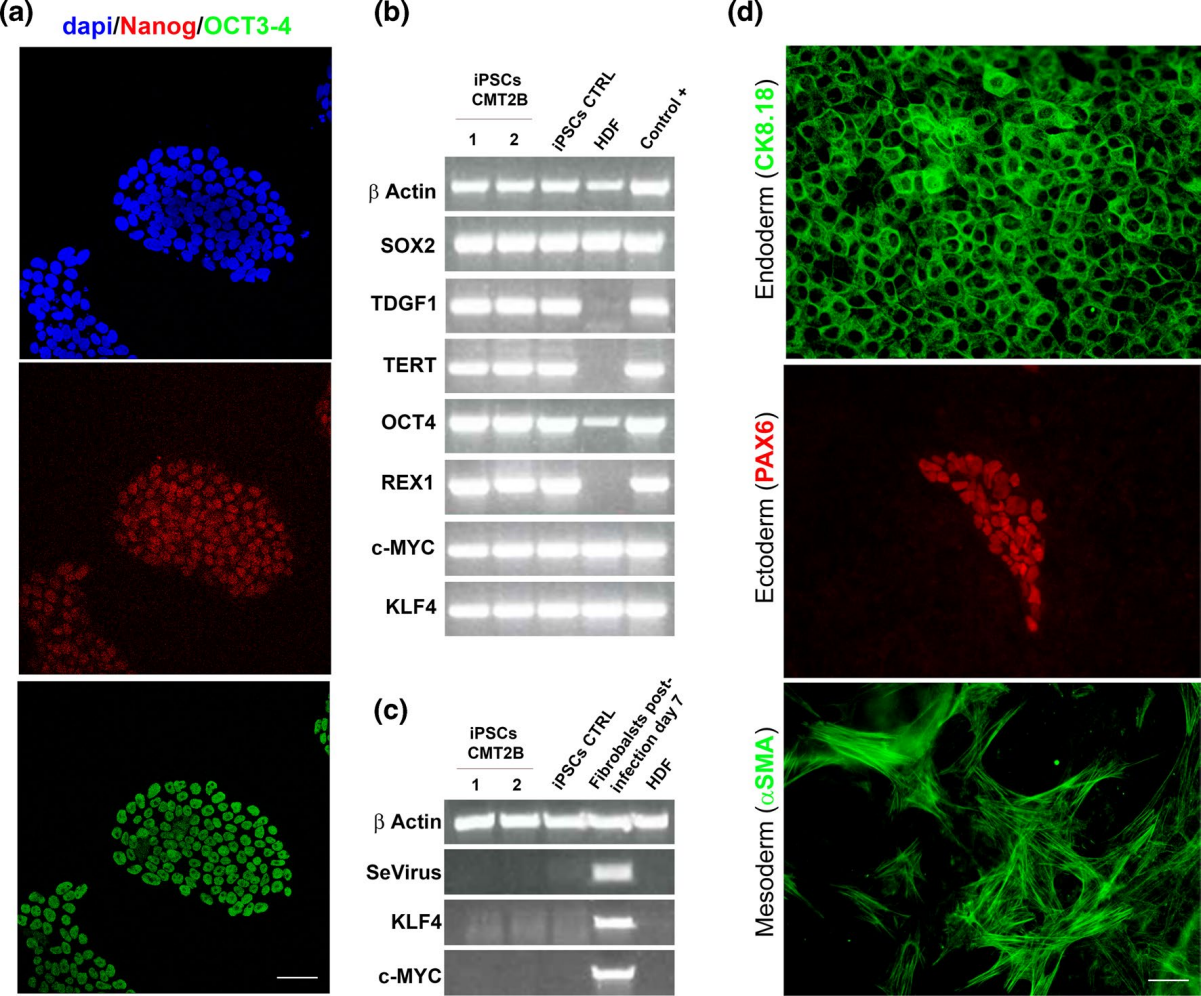
Expression of human embryonic stem cell markers and pluripotency of control and CMT2B hiPS cells. **a** Immunohistochemistry for embryonic stem (ES) cell markers of CMT2B iPS cells. Bars 50 µm. **b** RT-PCR analysis of ES cell-marker genes in human iPS cells, human dermal fibroblast (HDF) and previously derived iPS cell as positive control. Primers used for Oct3/4, Sox2, Klf4, and c-Myc specifically detect the transcripts from the endogenous genes, but not from the retroviral transgenes. **c** RT-PCR for expression of retroviral transgenes in human iPS cells, HDF, and HDF 7 days after the transduction with the four retroviruses as a positive control. **d** Immunohistochemistry of iPS cells spontaneously differentiated show markers for the three germ layers. Bars 50 µm

To confirm the data obtained in fibroblasts we decided to evaluate the abundance of lysosomal markers and lysosomal functionality in iPSC-derived sensory neurons from two CMT2B patients carrying the RAB7^V162M^ mutation compared to two healthy individuals. The iPS cells obtained from CMT2B patients and controls showed the expression of the expected markers of undifferentiated ES cells (Fig. 7a–c), as well as pluripotent differentiation capacity into the three germ layers in vitro and in vivo (Fig. 7d, Supplemental Fig. 1).

**Fig. 8:**
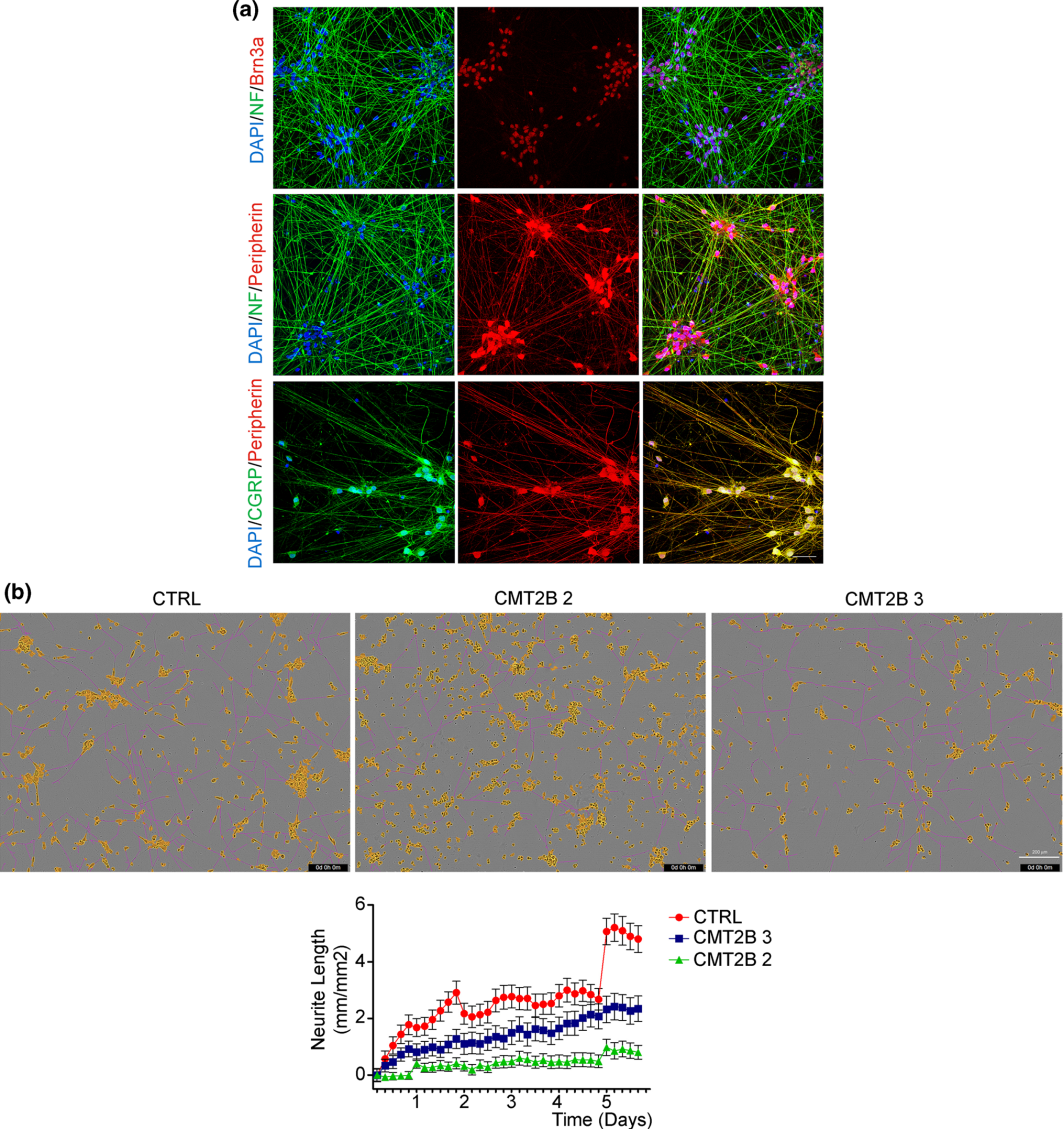
CMT2B peripheral sensory neurons show inhibited neurite outgrowth. **a** Control iPS cells were differentiated in peripheral sensory neurons and analyzed by immunohistochemistry at 17 days of differentiation for Brn3a, Neurofilaments, Peripherin and CGRP. Bars 50 µm. **b** Phase contrast images of iPSCs-derived sensory neurons from control (CTRL) and CMT2B patients carrying the RAB7^V162M^ mutation (#2 and #3) 6 days after plating. The IncuCyte automated acquisition and analysis show the extension of neurites growth (pink). Neuron cell body clusters are marked in yellow. Bar 200 µm. Automated quantification of neurite length for a time window of 6 days after plating. Neurite length data were collected in units of mm of total neurites detected/mm^2^ and all data are expressed relative to the respective day 0 of plating. Each group is represented by as mean ± SEM (*n* = 32 image)

iPSC-derived sensory neurons were grown on Matrigel-coated coverslips and showed normal axon network formation and expected markers of sensory dorsal root ganglia neurons (Fig. 8a). Previous data indicated that expression of CMT2B-causing RAB7 mutant proteins caused inhibition of neurite outgrowth [40, 41]. To assess neurite outgrowth in iPSCs-derived sensory neurons from control and CMT2B patients, the young neurons were plated and analyzed using IncuCyte^®^ S3 Live-Cell Analysis System for 6 days. Interestingly, CMT2B neurons showed reduced neurite extensions as compared to controls (Fig. 8b), confirming previous results obtained by transient expression of RAB7 mutant proteins.

**Fig. 9:**
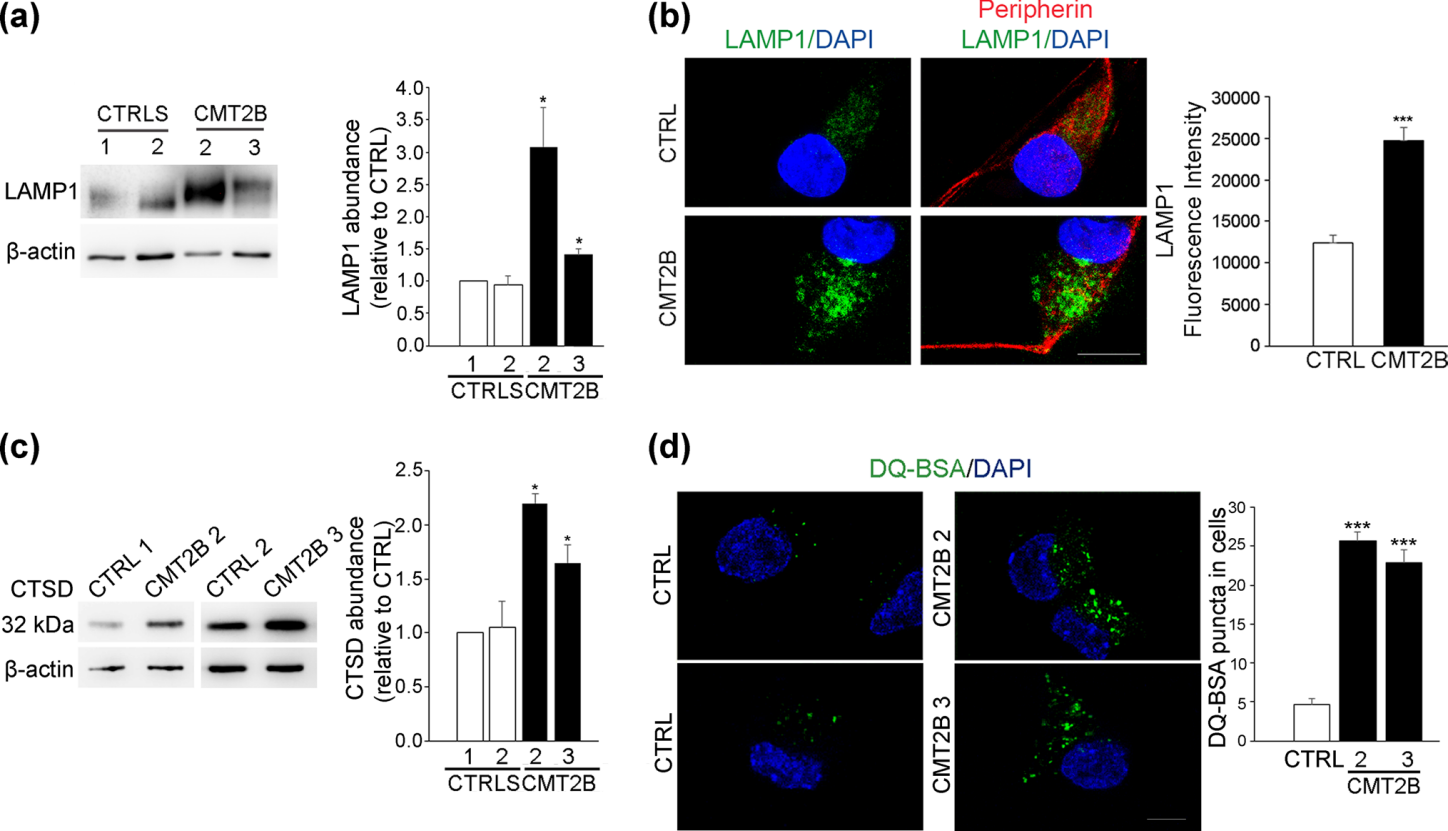
Lysosomal activity in iPSC-derived sensory neurons. **a** Lysates of neurons derived from controls and CMT2B fibroblasts carrying the RAB7^V162M^ mutation (patient 2 and 3) were subjected to immunoblotting using anti-LAMP1 and anti-β-actin antibodies. Intensities of bands were measured by densitometry and normalized against β-actin. Data represent the mean ± SEM of three experiments. Statistical analysis was performed using Student’s *t* test with control neurons as referring sample. *p < 0.05. **b** Control and CMT2B neurons from patient 2 were fixed and immunolabeled with anti-LAMP1 and anti-peripherin antibodies followed by Alexa488- and Alexa568-conjugated secondary antibody respectively while nuclei were stained with DAPI. Bars 10 µm. LAMP1 intensities were measured using ImageJ and Corrected Total Cell Fluorescence (CTCF) was calculated. Data represent the mean ± SEM of at least 50 cells analyzed. Student’s *t* test was used for statistical analysis. ***p < 0.001. **c** Lysates of neurons derived from controls and CMT2B fibroblasts (patient 2 and 3) were subjected to immunoblotting using anti-cathepsin D and anti-β-actin antibodies. Intensities of bands were measured by densitometry and normalized against β-actin. Data represent the mean ± SEM of three experiments. Statistical analysis was performed using Student’s *t* test with control neurons as referring sample. *p < 0.05. **d** Control and CMT2B neurons from patient 2 and 3 were treated with DQ-BSA for 24 h, then fixed, labeled with DAPI and observed on a confocal microscope. Bars 10 µm. DQ-BSA puncta in cells were measured using ImageJ. Data represent the mean ± SEM of at least 50 cells analyzed. For DQ-BSA quantification, Student’s *t* test was used for statistical analysis. ***p < 0.001

When we investigated in these cells late endosomal and lysosomal markers, we found that CMT2B neurons showed a twofold higher expression of LAMP1, confirming previous data obtained on CMT2B skin fibroblasts (Fig. 9a). We then performed immunofluorescence analysis to evaluate distribution and abundance of LAMP1-positive organelles and we observed a similar distribution in the perinuclear region of LAMP1 in the control and CMT2B neurons. However, CMT2B neurons showed a stronger expression of LAMP1 that was quantified confirming the data obtained by Western blotting and indicating an increase of about two times (Fig. 9b). We next investigated the abundance of the mature form of cathepsin D and we found an increase in CMT2B patients, similar to what we observed in fibroblasts (Fig. 9c). As higher cathepsin D maturation correlates with higher lysosomal activity, we performed a DQ-Red BSA assay. As expected, CMT2B neurons display higher fluorescent DQ-BSA staining than control neurons indicating higher lysosomal activity. Quantification of DQ-Green BSA puncta revealed an increase of about fivefold (Fig. 9d).

Altogether, these results indicate that iPS-derived neurons from CMT2B patient show higher lysosomal activity.

## Discussion

**Fig. 10:**
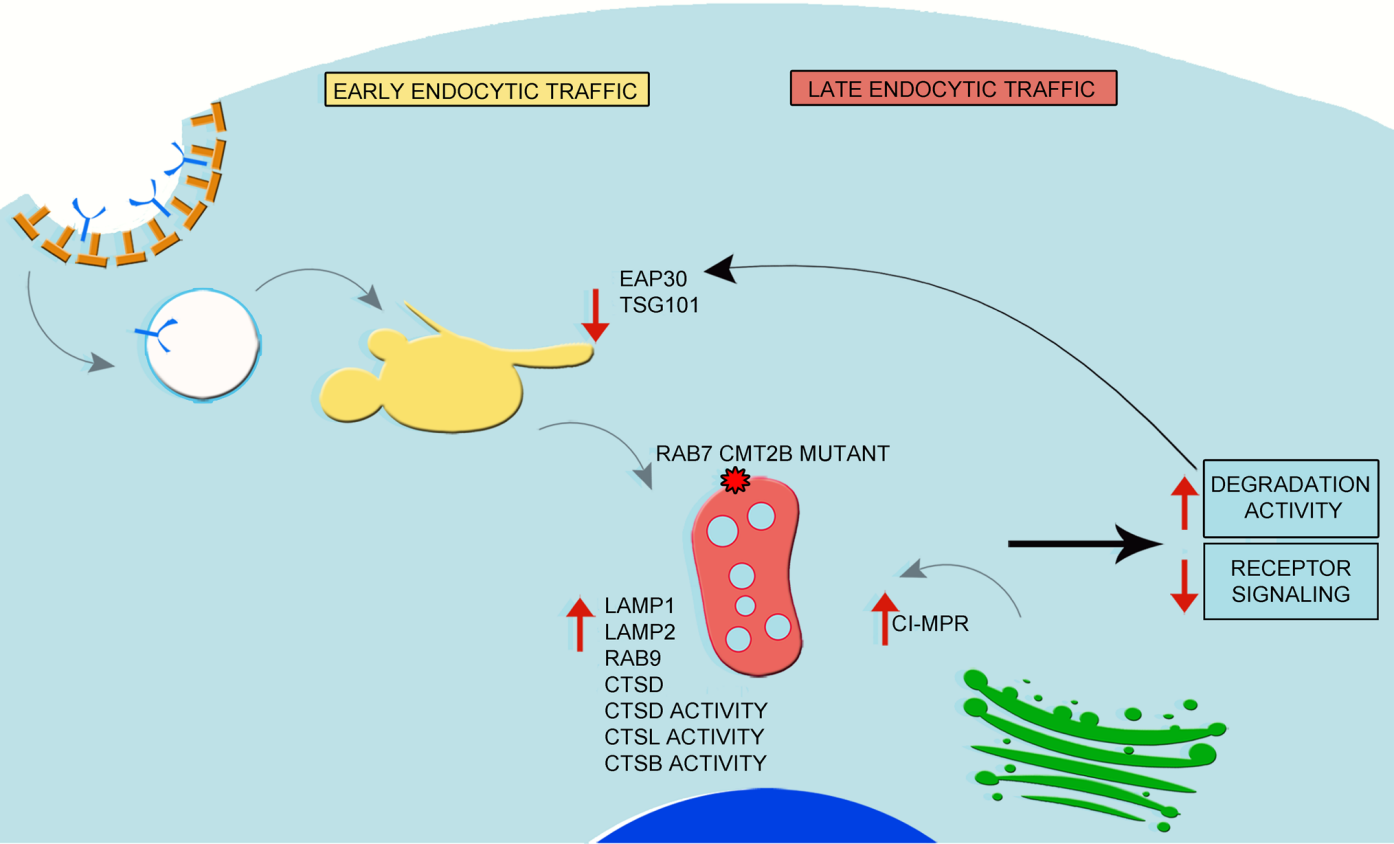
Model of the Impact of the CMT2B-causing RAB7 mutant protein on the endocytic pathway. In CMT2B the presence of RAB7^V162M^ causes an increase of late endosomal proteins and enzymes as well as of lysosomal activity. This, in turn, activates a feedback control mechanism demonstrated by the decrease of ESCRT proteins. Because of the presence of the RAB7 mutant, CMT2B cells fail to respond to this regulatory mechanism, showing higher lysosomal activity

In this study, we demonstrate that late endocytic traffic is altered in CMT2B compared to control cells. In particular, we show that RAB9 and CI-MPR as well as lysosomal proteins such as LAMP1, LAMP2 and cathepsin D are more abundant in these cells and that lysosomes, more numerous in CMT2B cells compared to control, display increased activity (Fig. 10). Indeed, the activity of three lysosomal enzymes (cathepsin B, D and L) is increased as well as degradation of EGFR and DQ BSA (Fig. 10). These data are consistent with several studies demonstrating that alterations of endocytic traffic induce neurodegeneration [11, 66]. In fact, upregulation of RAB proteins involved in endocytic traffic occurs during the progression of a number of neurodegenerative disorders including Alzheimer’s disease [67–70]. Furthermore, increased transport to endosomes of proteases, such as cathepsin B and L, coupled with higher expression of CI-MPR, was shown in Alzheimer’s disease [68, 71]. Indeed, in neurodegenerative disorders, increased endocytic flux induces abnormal degradation of signaling complexes, impairing neurotrophin receptor signaling that, in turn, could be responsible for neurodegeneration.

We also found that, although the expression of early endocytic RABs is not altered in CMT2B cells, EAP30 (ESCRT-II member) and TSG101 (ESCRT-I member) levels were strongly reduced (Fig. 10). The reduction of ESCRT components in CMT2B cells is consistent with previously reported cargo-dependent degradation of ESCRT components [72–74]. Indeed, it was shown that ESCRT-I components are delivered together with the cargo to lysosomes, where they are degraded [74]. Thus, lower levels of TSG101 are indicative of increased utilization. Therefore, our data on increased lysosomal activity and increased EGFR degradation are in agreement with lower abundance of ESCRT proteins. Furthermore, it has been demonstrated that one of the RAB7 mutant causing CMT2B, the RAB7^K157N^, interacts weakly with a subunit of the retromer complex that controls endosome-to-Golgi retrieval of CI-MPR receptor, thus reducing the efficiency of endosomal protein sorting [75]. Therefore, increased expression of CI-MPR in CMT2B cells could be due to a compensatory mechanism to counteract the presence of the RAB7^V162M^ mutant protein.

We observed increased degradative activity in CMT2B cells. Our data are in agreement with previous findings demonstrating that CMT2B-causing RAB7 mutants show increased interaction with ORP1L (cholesterol sensor oxysterol-binding protein-related protein 1L), and RILP (Rab-interacting lysosomal protein), two RAB7 effector proteins controlling transport of endosomes from the periphery of the cell toward the MTOC (MicroTubule Organizing Center) during endosome maturation [34]. Accordingly, it was shown that the expression of CMT2B-causing mutants restores EGFR degradation that was inhibited after RAB7 silencing [35, 36]. Moreover, higher lysosomal activity and increased degradation of EGFR in CMT2B cells suggest increased degradation also of other signaling receptors and consequent inhibition of signaling. Notably, expression of RAB7 mutant proteins affects the axonal transport by modifying trafficking and signaling of NGF (nerve growth factor) and its receptor, TrkA [76]. Importantly, it was also demonstrated that CMT2B-causing RAB7 mutants cause a reduction of TrkA surface level, which was hypothesized to be the consequence of premature degradation of TrkA induced by lysosomal activity [76]. In fact, hyperactivation of degradation within axons could induce a premature termination of TrkA signaling, with consequent inhibition of retrograde signaling, possibly contributing to axonal degeneration [76]. Finally, it was reported that increased endocytic flux in neurons induces neurodegeneration in Alzheimer’s disease, as a consequence of reduced neurotrophin receptors expression and signaling, [67, 69]. Thus, we hypothesize that the increased lysosomal activity could affect signaling because of premature degradation of signaling receptors, thus contributing to the axonal degeneration occurring in CMT2B disease.

We also observed an increased migration of CMT2B fibroblasts, though EGFR degradation was higher in these cells. These results may appear contradictory, as it was shown that the downstream signaling pathways of numerous receptor tyrosine kinases, including EGFR, are involved in the regulation of cell motility [77, 78]. Indeed, extracellular-regulated kinase (ERK), Jun kinase and tumor protein (p)38 affect various cell functions, including migration [79]. Also phosphatidylinositol-3 kinase (PI3K) controls cell motility through the activation of protein kinase B (Akt) and other targets [80, 81]. However, cell migration does not exclusively depend on EGFR signaling. In fact, it was demonstrated that inhibition of EGFR activation in tumor cells leads to activation of a β1 integrin pathway that promotes migration, undermining EGFR blockade [82]. Furthermore, there are many mechanisms that promote migration independently of Akt, and one of the most important is the remodeling of the actin cytoskeleton mediated by RAC1 [83, 84]. In fact, it was found that β1 integrins locally activate RAC1 [85]. In CMT2B cells, we found an increased RAC1 activation, while we did not detect any difference in RAC1 total protein amount. Therefore, our data are in agreement with previous findings showing the existence of alternative pathways that regulate cell motility. Furthermore, it was previously reported that RAC1 promotes MMP-2 activation [63] and we observed higher activation of this gelatinase in CMT2B patients. Thus, a decrease in EGFR signaling due to its higher degradation could trigger β1 integrin pathway that leads to RAC1 and MMP-2 activation, causing increased migration of CMT2B cells in an EGFR-independent manner.

We previously found that the expression of CMT2B-causing RAB7 mutants negatively affects the role of RAB7 in autophagy [43]; in particular, in CMT2B fibroblasts we observed a reduced localization of RAB7 on autophagosomes after autophagy induction, a decreased number of LC3B-positive vesicles and a reduced autophagic flux, demonstrated by bafilomycin A_1_ treatment [43]. We also established that the expression of the RAB7^V162M^ does not affect autophagosomal biogenesis, but it only alters later phases of autophagosome maturation [43]. Considering that RAB7 controls fusion of autophagic vacuoles with late endosomes and lysosomes, the present data, showing increased expression of lysosomal membrane proteins and enzymes and increased lysosomal activity, seem to be in contrast. However, it was reported that thapsigargin distinguishes membrane fusion in the late stages of the two pathways, endocytosis and autophagy, indicating that the two pathways, although sharing many components of the membrane traffic machinery, can be independently regulated [86, 87]. In addition, CMT2B mutations do not generate classical gain or loss of function RAB7 mutants. It was reported that RAB7 mutants would cause the disease due to the misregulation of native RAB7 activity [34]. Indeed, these mutant proteins display a higher nucleotide *K*_off_ and thus they tend to release nucleotides (both GTP and GDP) earlier as compared to the wt protein [34–36]. The *k*_off_ is higher for GDP than for GTP and, considering that GTP concentration in the cell is approximately one order of magnitude higher than GDP, these mutants are mainly in the GTP-bound form, as each time that they release GDP prematurely they have a higher probability to bind GTP. However, due to the increased *K*_off_ for GTP compared to the wt protein, they also tend to release GTP prematurely and this affects negatively the GTPase activity per binding event [34–36]. Thus, these mutant proteins are dysfunctional, as they have unregulated nucleotide exchange and activation [34]. They can stimulate a process as they are GTP bound but, at the same time, if the reaction controlled by RAB7 requires being stimulated by RAB7-GTP for a certain amount of time, premature release of GTP could block the controlled process. Hence, depending on the kinetic requirements of the regulated process, these mutant proteins could behave as active or inhibitory mutants. These considerations may explain the apparently conflicting data present in the literature. Indeed, for instance, in the Drosophila model of the disease, it was established that neurodegeneration was due to a partial loss of function of RAB7, while studies on zebrafish showed that CMT2B-causing RAB7 mutations induce defects in axon growth, similarly to the constitutively active form of RAB7, thus suggesting that defects observed in the neuropathy are caused by excess of RAB7 activity [37, 38, 88]. It is also worth noting that it was demonstrated that even though RAB7 mutants are inefficiently recruited to the endosomes, endosomal maturation is not impaired [38]. Moreover, it was shown that a dominant negative mutant of RAB7 induces endosomal accumulation of TrkA, enhancing TrkA signaling and neurite outgrowth, while CMT2B-causing RAB7 mutants inhibit this process, again behaving similarly to the constitutively active mutant [13, 40]. Thus, RAB7 mutants are dysfunctional and distinct regulatory processes are differently affected by their presence. Accordingly, CMT2B-causing RAB7 mutants impact differently on autophagy and endocytosis.

Several CMT genes affect the autophagy pathway at different stages, suggesting that autophagy may represent a common pathomechanism in inherited peripheral neuropathies, including CMT1A and CMT1E in which mutations in PMP22 (peripheral myelin protein 22) disrupt the initiation of autophagy [89]. However, despite these premises, autophagy modulation as a treatment for hereditary neuropathies remains elusive [89]. Our present data widen the current knowledge about pathomechanisms of CMT2B, demonstrating changes in the expression of different proteins along the endocytic degradative pathway that could become targets to revert the disease phenotype.

This study has been performed on skin fibroblasts from three CMT2B patients with the RAB7^V162M^ mutation and on iPS-derived sensory neurons of two patients. Although these data are consistent with previous and present data obtained on continuous cell lines transfected with plasmids for expression of CMT2B-causing RAB7 mutant proteins, the number of patients is very limited, due to the fact that CMT2B is a rare form of a rare disease. Furthermore, the three patients were from a single family and there may be modifier variants in this family. Thus, it is fundamental to expand in the future the number of patient cell lines to strengthen our findings.

## Electronic supplementary material

Below is the link to the electronic supplementary material.

Supplementary file1 (DOCX 3002 kb)
