# Supplementary material for: Alteration of the late endocytic pathway in Charcot–Marie–Tooth type 2B disease
Source: Cell Mol Life Sci. 2020 Apr 13;78(1):351–72. doi: 10.1007/s00018-020-03510-1 (PMC7867545; doi:10.1007/s00018-020-03510-1)
Supplement: Supplementary file 1 — Supplementary file1 (DOCX 3002 kb) [file 18_2020_3510_MOESM1_ESM.docx]

**Supplementary material**

**Alteration of the late endocytic pathway in Charcot-Marie-Tooth type 2B disease**

**Roberta Romano^1^, Cristina Rivellini^2^, Maria De Luca^1^, Rossana Tonlorenzi^2^, Raffaella Beli^1^, Fiore Manganelli^3^, Maria Nolano^4^, Lucio Santoro^3^, Eeva-Liisa Eskelinen^5^, Stefano C. Previtali^2^, Cecilia Bucci^1*^**

^1^Department of Biological and Environmental Sciences and Technologies (DiSTeBA), University of Salento, Lecce, Italy

^2^Institute of Experimental Neurology (INSPE), San Raffaele Scientific Institute, Milan, Italy

^3^Department of Neurosciences, University of Naples “Federico II”, Naples, Italy

^4^Salvatore Maugeri Foundation, Institute of Telese Terme, Benevento, Italy

^5^Institute of Biomedicine, University of Turku, Turku, Finland, and Molecular and Integrative Biosciences Research Programme, University of Helsinki, Helsinki, Finland

*** Correspondence:**Cecilia Bucci
cecilia.bucci@unisalento.it

**Figures and Tables:**

**Supplemental Figure 1 iPSC characterization and DNA sequence analysis of RAB7. (a)** Phase-contrast images of control and CMT2B iPS cells colonies. Bars = 50 µm. **(b)** Haematoxylin and eosin staining of teratoma derived from CMT2B iPS cells. iPS cells generate tumor composed by three germ leyers: (A) epithelial cells in glandular structure, (B) globular epithelial cell, (C) neuroectodermal structure, (D) pigmentated retinal cells, (E) cartilage, and (F) mesodermal cells including smooth muscle cells; Bars = 50 µm. **(c)** RT-PCR analyses of various differentiation markers for the three germ layers. **(d)** Electropherogram showing the c.484 G > A sequence variation in part of exon 4, resulting in the Val162Met missense mutation in patient 2. The corresponding genomic sequence of a control is shown on the top. DNA sequencing was performed by Eurofins Genomics, Ebersberg, Germany.


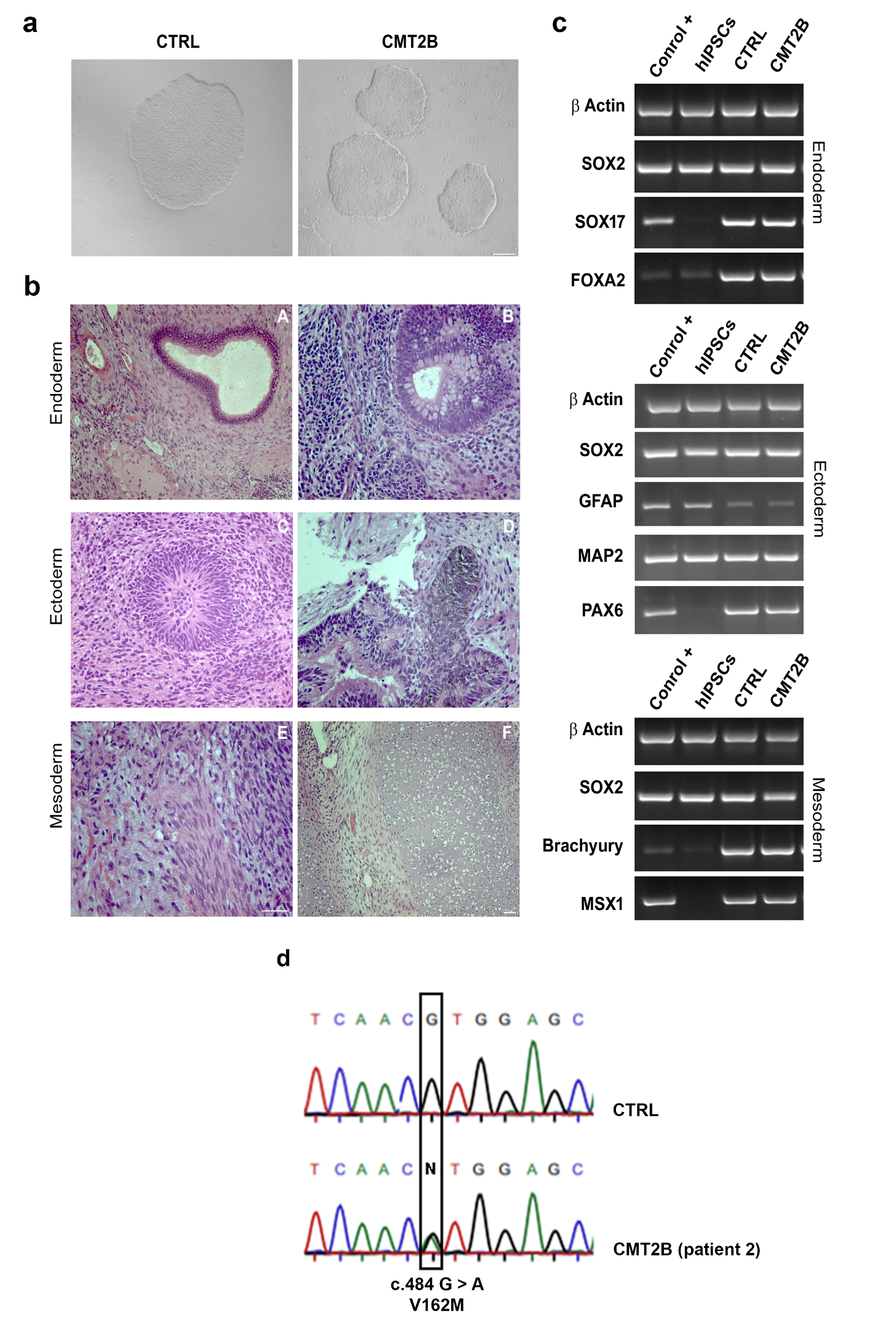


**Supplemental Table 1 Programme used to differentiate iPSCs in sensory neurons**

| Day #0 | KSR medium +SMAD inhibitors (SB+LDN) |
| --- | --- |
| Day #1 | KSR med +(SB+LDN) |
| Day #2 | KSR med+(SB+LDN)+(CHIR+SU+DAPT) |
| Day #3 | KSR med+(SB+LDN)+(CHIR+SU+DAPT) |
| Day #4 | KSR med+( SB+LDN)+(CHIR9+SU+DAPT) |
| Day #5 | 75% KSR med/25% N2B27 + (SB+LDN)+(CHIR+SU+DAPT) |
| Day #6 | 75% KSR med/25% N2B27 + (CHIR+SU+DAPT) |
| Day #7 | 50% KSR med/50%N2B27 +(CHIR+SU+DAPT) |
| Day #8 | 50% KSR med/50%N2B27+(CHIR+SU+DAPT) |
| Day #9 | 25% KSR med/ 75% N2B27) + (CHIR+SU+DAPT) |
| Day #10: | 25% KSR med/ 75% N2B27 + (CHIR+SU+DAPT) |
| Day #11 | N2B27+(CHIR+SU+DAPT) |
| Day #12 | N2B27+(CHIR+SU+DAPT) |
| Day #13 | Neural diff. medium+CHIR99021 |
| Day #14 | Neural diff. medium+CHIR99021 |
| Day #15 | Neural diff. medium +CHIR99021 |
| From day #16 onwards | Neural diff. medium |

**Supplemental Table 2 Primers used for RT-PCR**

| hβACTIN F | CAA CCG CGA GAA GAT CAC |
| --- | --- |
| hβACTIN R | AGG AAG GCT GGA AGA GTG |
| hSOX2 F | TTA CCT CTT CCT CCC ACT CCA G |
| hSOX2 R | GGG TTT TCT CCA TGC TGT TTC T |
| hTDGF1 F | TTT GCT CGT CCA TCT CGG G |
| hTDGF1 R | GCT CCT TAC TGT GCT GTA TCC C |
| hTERT F | CAG GAG CTG ACG TGG AAG ATG |
| hTERT R | GAA ACG TGG TCT CCG TGA CA |
| hOCT4-2 F | ATG CAC AAC GAG AGG ATT TTG A |
| hOCT4-2 R | CTT TGT GTT CCC AAT TCC TTC C |
| hREX1 F | CAG ATC CTA AAC AGC TCG CAG AAT |
| hREX1 R | GCG TAC GCA AAT TAA AGT CCA GA |
| hc-MYC F | CCA AAG TCC AAT TTG AGG CAG T |
| hc-MYC R | AGC AGA GGA GCA AAA GCT CAT T |
| hKLF4-2 F | ACC CAC ACA GGT GAG AAA CCT T |
| hKLF4-2 R | GTT GGG AAC TTG ACC ATG ATT G |
| hSOX17 F | CGC TTT CAT GGT GTG GGC TAA GGA CG |
| hSOX17 R | TAG TTG GGG TGG TCC TGC ATG TGC TG |
| hFOXA2 F | TGG GAG CGG TGA AGA TGG AAG GGC AC |
| hFOXA2 R | TCA TGC CAG CGC CCA CGT ACG ACG AC |
| hGFAP F | GGC CCG CCA CTT GCA GGA GTA CCA GG |
| hGFAP R | CTT CTG CTC GGG CCC CTC ATG AGA CG |
| hMAP2 F | CAG GTG GCG GAC GTG TGA AAA TTG AGA GTG |
| hMAP2 R | CAC GCT GGA TCT GCC TGG GGA CTG TG |
| hPAX6 F | ACC CAT TAT CCA GAT GTG TTT GCC CGA G |
| hPAX6 R | ATG GTG AAG CTG GGC ATA GGC GGC AG |
| hBrachyury F | GCC CTC TCC CTC CCC TCC ACG CAC AG |
| hBrachyury R | CGG CGC CGT TGC TCA CAG ACC ACA GG |
| hMSX1 F | CGA GAG GAC CCC GTG GAT GCA GAG |
| hMSX1 R | GGC GGC CAT CTT CAG CTT CTC CAG |
